# Interlocked Rotaxane Enables TADF with Distinct Excited-State Structural Relaxation

**DOI:** 10.1021/jacs.5c19031

**Published:** 2026-01-29

**Authors:** Chuan-Jing Lin, Kai-Hsin Chang, Chun-Yen Lin, Kuan-Hsuan Su, Chieh-Ming Hung, Yi-Hung Liu, Orion Shih, Ken-Tsung Wong, Pi-Tai Chou

**Affiliations:** † Department of Chemistry, 33561 National Taiwan University, Taipei 106319, Taiwan; ‡ National Synchrotron Radiation Research Center (NSRRC), Hsinchu 300092, Taiwan; § Institute of Atomic and Molecular Science, Academia Sinica, Taipei 106319, Taiwan

## Abstract

We present the first demonstration of a rotaxane-based thermally activated delayed fluorescence (TADF) exciplex, its unique excited-state structural relaxation and application in organic light-emitting diodes (OLEDs). The design employs a triazene cage (**Trz-cage**) as the host electron acceptor, threaded by a carbazole derivative with ethylene glycol ether chains serving as the guest donor, and capped at both ends with bulky triphenylmethane stoppers, thus forming the rotaxane exciplex, namely the charge-transfer **CT-Rotaxane**. The TADF nature of **CT-Rotaxane** is evidenced by microsecond-scale delayed fluorescence subject quenched by oxygen, a small singlet–triplet energy gap (Δ*E*_ST_ = 0.084 eV), and a fast reverse intersystem crossing rate of 9.8 × 10^5^ s^–1^ in toluene. Notably, the rotaxane TADF exciplex undergoes pronounced structural relaxation in both solution (τ ≈ 264 ps) and solid state (τ ≈ 177 ns), corroborated by combined quantum mechanical and molecular dynamics simulations. Importantly, the interlocked **CT-Rotaxane** enabled the fabrication of rotaxane-type OLEDs that delivered green electro-luminescence (EL) with a peak external quantum efficiency (EQE) of 7.23% at 263 cd m^–2^—surpassing the reference nonrotaxane **1@Trz-cage** and **TrMe@Trz-cage** exciplex OLEDs in efficiency and operational stability, respectively. These findings underscore mechanically interlocked TADF exciplexes as a promising strategy for optoelectronic applications.

## Graphical abstract

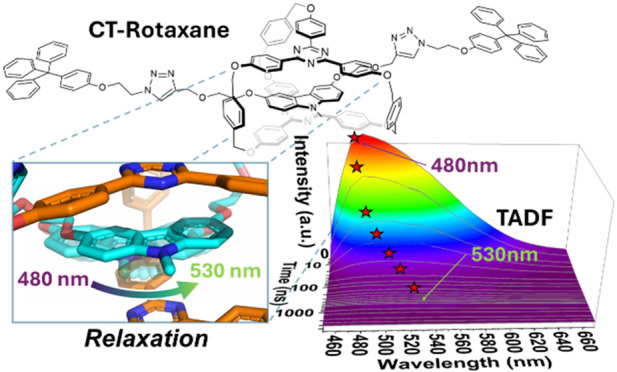

## Introduction

By designing electron donor (D)–acceptor (A) pairs with weak electronic coupling, the excited-state charge transfer can generate an exciplex in which the spatially separated charges facilitate thermally activated delayed fluorescence (TADF). Such systems have emerged as a promising strategy for achieving highly efficient organic light-emitting diodes (OLEDs).^1,2^ However, the precise D/A structural arrangements had long remained elusive. Only recently has the TADF exciplex structure been probed by time-resolved vibrational spectroscopy,^3^ and more conclusively, by the successful growth of TADF D/A cocrystals analyzed using X-ray diffraction.^4–12^ The TADF cocrystals were obtained through inclusion complexation between a cage-like electron acceptor, a triazene derivative (**Trz-cage** ^4^ see Scheme 1), and a series of electron donors, which unambiguously revealed the D–A structures, showing donor encapsulation within the cage. Unfortunately, because their formation is entropy-driven and endothermic, the D/A TADF cocrystals have a strong tendency to dissociate in solution, hindering further detailed dynamic studies. The weak D/A association also complicates device fabrication: during spin-coating, which requires solution-phase processing, the complexes are prone to dissociation, ultimately leading to instability under OLED operation. Weak donor–acceptor interactions are critical for promoting spatial charge separation and enabling TADF in exciplex systems. To suppress dissociation while preserving these essential interactions, a practical approach is to design D/A complexes incorporating kinetic stabilization. In this context, a rotaxane-type D/A complex capable of exciplex TADF represents an ideal platform. Motivated by this rationale, we undertook a comprehensive literature survey. In 2001, Swager and co-workers reported intramolecular exciplex green fluorescence from a rotaxane.^13^ More recently, Stoddart and co-workers described mechanically bond–induced exciplex fluorescence in an anthracene-based homo[2]­catenane^14^ and thermally controlled exciplex fluorescence in a dynamic homo[2]­catenane,^15^ but TADF was not assigned in either case. Zysman-Colman et al. subsequently demonstrated that a mechanical bond could tune intramolecular TADF emission in a rotaxane,^16^ yet this system did not involve an exciplex. To date, however, a mechanically interlocked (rotaxane) exciplex that unambiguously exhibits prominent TADF remains unreported.

**Scheme 1:**
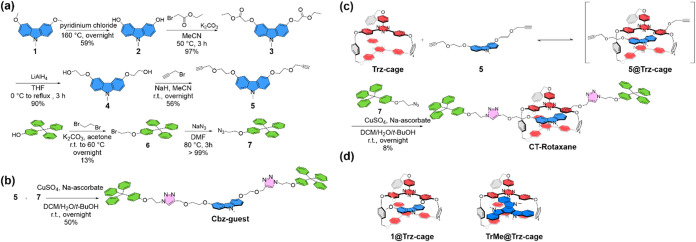
Synthetic Routes of (a) Donor and Stopper Units, (b) Thread (**Cbz-guest**), (c) **CT-Rotaxane**, and (d) The Structures of Reference Complexes **1@Trz-cage** and **TrMe@Trz-cage**

Herein, we report the design, synthesis, and structural characterization of a rotaxane-type TADF exciplex. The design strategy employs a triazene (**Trz**)-cage as the host electron acceptor, threaded by a linear carbazole-derived electron donor and terminated at both ends with bulky triphenylmethane stoppers. This architecture yields a rotaxane-type weak D/A complex (**CT-Rotaxane**) that features TADF behavior with small Δ*E*_ST_ arising from the intercomponent CT state. Importantly, the rotaxane TADF exciplex exhibits pronounced structural relaxation in both solution and solid state, as revealed by time-resolved emission spectral evolution and corroborated by quantum mechanical (QM)/molecular dynamics (MD) simulations. The kinetic stability of the **CT-Rotaxane** further enables, for the first time, the fabrication of rotaxane-type OLEDs and their unique structural relaxation properties. The results and their implications are presented in the following sections.

## Results and Discussion

### Syntheses and Characterization

Scheme 1 depicts the synthetic routs for the studied molecules. Detail of syntheses and characterization is elaborated in the Supporting Information (SI). In brief, the strategy is to design an ethynyl group-capped carbazole donor for the formation of exciplex-enabling supramolecular complex with the electron acceptor **Trz-cage**.^17^ Subsequently, an azide-functionalized sterically hindered molecule was introduced as the stopper to construct the rotaxane through a click reaction. For the synthesis of the donor component, 3,6-dimethoxy-9-methyl-9*H*-carbazole (**1**) was used as the starting material (Scheme 1a). The methyl groups of **1** were removed by using pyridinium chloride to afford compound **2**,^18^ which subsequently underwent an S_N_2 reaction with ethyl bromoacetate to yield the diester compound **3**.^17^ Reduction of **3** with LiAlH_4_ led to the diol compound **4**, followed by an S_N_2 reaction with propargyl bromide, afforded compound **5**.^19^ For the preparation of the stopper fragment, 4-tritylphenol was used as the starting material. Dibromoethane was employed to extend the carbon chain to yield compound **6**, which subsequently underwent substitution with sodium azide to give compound **7**.^20^ A model click reaction using compounds **5** and **7** was subsequently carried out. The click reaction proceeded smoothly in the presence of CuSO_4_ and sodium ascorbate,^21^ yielding the desired thread (**Cbz-guest**) in 50% isolated yield (Scheme 1b). This result validated the feasibility of the click strategy, which was then applied to the preparation of desired rotaxane system. Donor **5** was then introduced into the **Trz-cage** through a host–guest equilibrium process. A 2:1 mixture of compound **5** and the **Trz-cage** in CH_2_Cl_2_ was heated to promote complex formation. Upon slow solvent evaporation at 60 °C, a preorganized intermediate complex was obtained. The subsequent click reaction, performed in the presence of compound **7**, successfully trapped the donor within the **Trz-cage** via mechanical interlocking, yielding the rotaxane structure (Scheme 1c).

High-resolution mass spectrometry (HRMS) analysis of **CT-Rotaxane** showed a molecular ion peak at *m*/*z* = 2208.9, consistent with the expected molecular weight (Figure S2). The ^1^H NMR spectrum revealed that the originally symmetric chemical environment of the **Trz-cage** became asymmetric upon thread incorporation. In the 2D ^1^H–^1^H COSY spectrum (Figure S1), a series of well-defined cross-peaks appeared in the 3.0–5.0 ppm region, corresponding to the CH_2_–CH_2_ segments of the thread linker, exhibiting a characteristic AB-type coupling pattern. In the aromatic region (6.5–8.5 ppm), multiple sets of cross-peaks were observed, indicative of ortho coupling between aromatic protons. These correlations further support the peak assignments in the 1D ^1^H NMR spectrum, and collectively confirm that the thread is mechanically interlocked within the **Trz-cage**.

Single crystals of **CT-Rotaxane** were successfully obtained by slow diffusion of methanol into a solution of **CT-Rotaxane** in ethyl acetate. As shown in Figure 1a, X-ray crystallographic analysis reveals that the central carbazole unit is not fully encapsulated by the **Trz-cage**; only one of its phenylene rings is inserted into the cavity. The distances between carbazole and **Trz-cage** are approximately 3.6 and 3.7 Å, indicating π–π stacking interactions (Figure 1b). Obviously, the thread (**Cbz-guest**) adopts a bent conformation rather than a linear geometry. This distortion is presumed to arise from noncovalent interactions between the two stopper units, as well as specific interactions between the thread and the pillar components of the **Trz-cage** framework. The crystal unit cell contains four **CT-Rotaxane** molecules (Figure S3), among which multiple intermolecular π–π and C–H···π contacts are observed between adjacent rotaxanes, further supporting the mechanically interlocked architecture and highlighting the contribution of supramolecular packing to structural stability in the crystal state.

**Figure 1:**
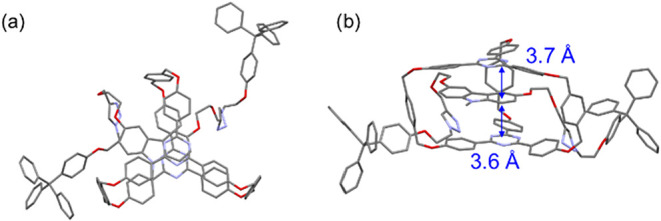
(a) Top view and (b) side view of the X-ray analyzed crystal structure of **CT-Rotaxane**.

### Absorption and Emission Spectroscopy/Dynamics

Figure 2a presents the absorption and photoluminescence spectra of the individual **Cbz-guest**, **Trz-cage**, and **CT-Rotaxane**. Because **CT-Rotaxane** is nearly insoluble in nonpolar solvents such as cyclohexane, its absorption spectrum was measured in weak polar solvents such as toluene. The **Trz-cage** and **Cbz-guest** show absorption edges at ∼350 nm and ∼400 nm, respectively, whereas their **CT-Rotaxane** complex exhibits a red-shifted absorption extending to ∼450 nm (inset of Figure 2a). The **CT-Rotaxane** exhibits a broad, structureless emission band centered at ∼505 nm in toluene. The excitation spectrum monitored at this emission maximum matches the corresponding absorption spectrum (Figure S10), confirming that the emission indeed originates from the **CT-Rotaxane**. Notably, this 505 nm emission is markedly red-shifted compared to the intrinsic fluorescence of the **Cbz-guest** (400 nm) and **Trz-cage** (380 nm) components (Figure 2a). Moreover, the emission displays a pronounced bathochromic shift with increasing solvent polarity—from toluene to dichloromethane (DCM) and acetonitrile (ACN)—while the absorption spectra remain unaffected (Figure S8b). Such a large Stokes shift provides compelling evidence for through-space charge transfer between the donor (**Cbz-guest**) and acceptor (**Trz-cage**) units, resulting in exciplex-type emission. This interpretation is consistent with the electronic structure revealed by natural transition orbital (NTO) analysis of the S_1_ state (Figure S23). The S_1_ transition is characterized by a spatial separation between the HONTO (hole), which is localized on the **Cbz-guest** and the LUNTO (electron), which resides on the **Trz-cage**, with a calculated hole–electron centroid distance of 3.27 Å. This distance is highly consistent with the intermolecular π-π stacking distance (3.6–3.7 Å) observed in the crystal structure, supporting that the observed exciplex-type emission originates from a through-space charge-transfer process across the interlocked donor–acceptor interface.

**Figure 2:**
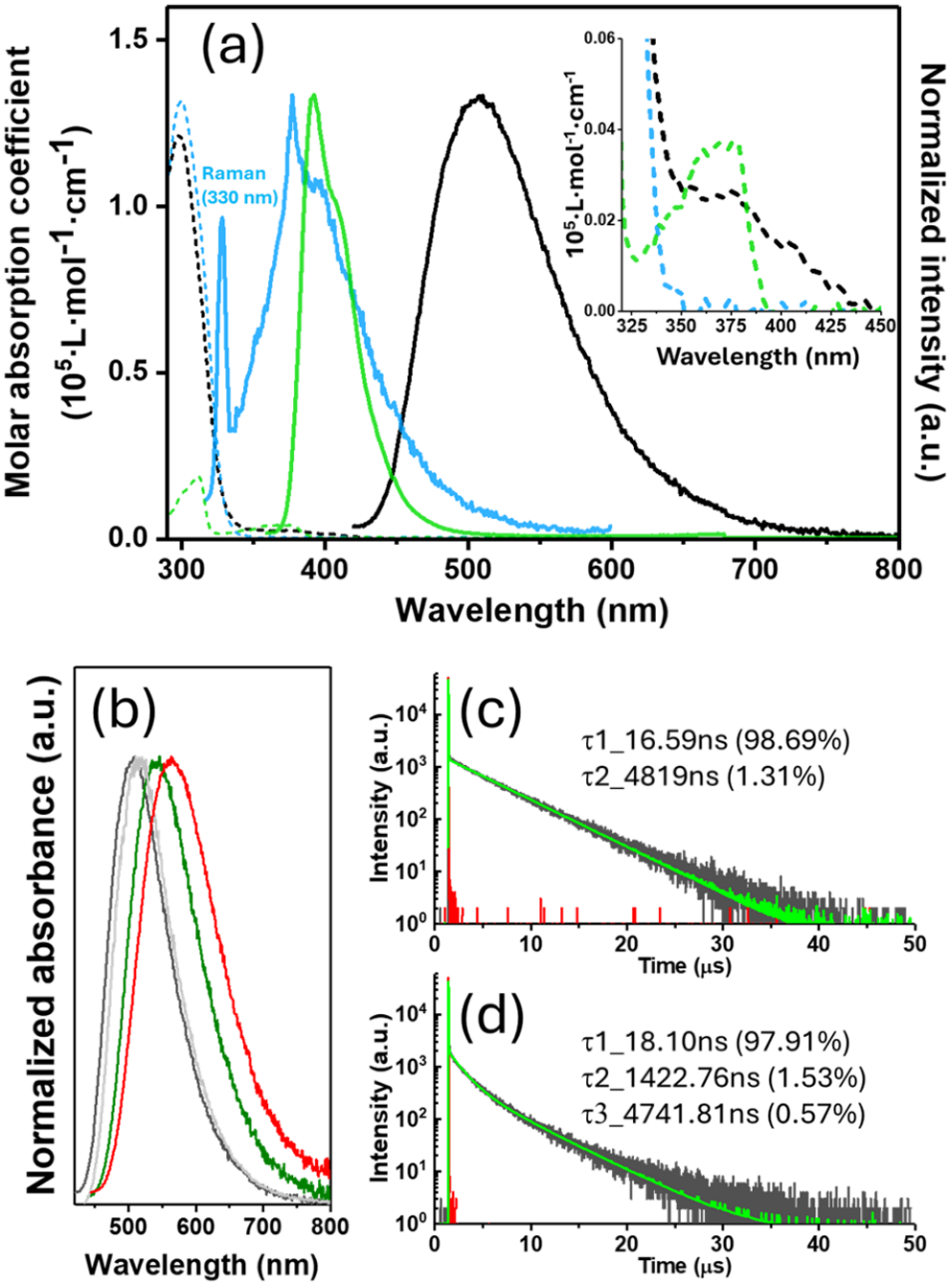
(a) Molar absorption coefficients (dashed lines) and emission spectra (solid lines) of **CT-Rotaxane** (black), **Trz-cage** (sky blue, the peak at ∼330 nm arises from Raman scattering upon 300 nm excitation), and **Cbz-guest** (green) in toluene. The inset enlarges the molar absorption spectrum. (b) The emission spectra of **CT-Rotaxane** in toluene (black), DCM (olive), ACN (red), and the solid powder (gray). The time-resolved profiles of **CT-Rotaxane** in toluene (degassed) (c) and solid powder (d). The emission dynamics profile was monitored at 505 nm. (The prompt fluorescence dynamics profiles are well reproduced by the fitted curves, as shown in Figure S21).

The exciplex emission of **CT-Rotaxane**, arising from the spatially separated **Cbz-guest** and **Trz-cage** units, is expected to exhibit TADF behavior.^4^ As shown in Figure S12a, the 505 nm exciplex emission in toluene displays a pronounced enhancement in intensity upon degassing compared with aerated conditions; the photoluminescence quantum yield (PLQY) increases from ∼3% in aerated toluene to ∼22% after degassing, a value comparable to the 23% measured for **CT-Rotaxane** in the solid film (amorphous). In this study, temperature-dependent measurements were not employed to estimate Δ*E*_ST_ from the temperature-dependent decay lifetimes described in eq (S34) in SI.^23,24^ This is because **CT-Rotaxane** undergoes strong structural relaxation in the solution phase when it is not frozen, leading to multiple decay components that obscure the separation of TADF dynamics from structural-relaxation processes. Instead, Δ*E*_ST_ was determined from the difference between the onset energies of the fluorescence and phosphorescence spectra at 77 K, where the solvent matrix is completely frozen (Figure S17), giving a value of 0.084 eV. The combination of strongly O_2_-quenched emission and the small Δ*E*_ST_ derived from steady-state measurements provides compelling evidence for the TADF behavior of the exciplex emission in **CT-Rotaxane**.

Further insights into the emission properties were obtained by time-resolved fluorescence spectroscopy. Time-correlated single-photon counting (TCSPC) technique was employed for these measurements: the nanosecond-to-microsecond regime was recorded on a fluorescence spectrometer (FLS-980, Edinburgh Instruments Ltd.) with a detection limit of ≈200 ps. For time-resolved emission measurements in the picosecond–nanosecond regime, we employed a TCSPC system equipped with a femtosecond laser excitation source (380–420 nm, 120 fs) and a microchannel plate photomultiplier tube (MCP-PMT) detector, providing an overall instrument response of ≈20 ps. The TADF and excited-state structural relaxation dynamics of **CT-Rotaxane** in solution were recorded under degassed conditions using the freeze–pump–thaw method to eliminate O_2_ interference. Figures 2c and S12c,d reveal characteristic TADF behavior of **CT-Rotaxane** in toluene, showing a biexponential decay consisting of a prompt lifetime of ∼16.6 ns and a long-lived component of ∼4.8 μs. Kinetic fitting based on coupling reaction dynamics (see SI) yielded intersystem crossing rate constant *k*_ISC_ of 4.8 × 10^7^ s^–1^ and reverse intersystem crossing rate constant *k*_RISC_ of 9.8 × 10^5^ s^–1^. Pertinent data are summarized in Table S2 of SI. Upon monitoring at the early time domain based on the 20 ps response-time TCSPC, interestingly, we found that the emission relaxation dynamics are wavelength dependent within 1 ns. As shown in Figure 3a, upon monitoring at blue region of the emission, e.g., 440 nm, the relaxation time profile reveals a fast decay component of ∼320 ps, followed by a ∼16 ns prompt emission (vide supra). In sharp contrast, we observed a rise component of ∼150 ps (see also Figure S11) and a ∼16 ns decay when monitoring at the emission red edge of ∼650 nm. To gain more insight, we then carefully measured the relaxation dynamics throughout the entire emission region. The emission intensities at selected time intervals were then reconstructed as a function of wavelength, thereby generating the emission spectral–temporal evolution map.^25^ The results depicted in Figure 3b clearly show a time-dependent shift of the peak wavelength continuously within tens to hundreds picoseconds; instead of switching between two distinct emissive states. Furthermore, this unusually long relaxation time scale readily distinguishes it from typical solvent relaxation that generally occurs within 5–6 ps in toluene.^26^ Therefore, the kinetic precursor-successor relationship observed in Figure 3a cannot be attributed to solvent relaxation. Alternatively, the results are rationalized by weak host–guest interaction for **CT-Rotaxane** in the ground state. The loose rotaxane structure, upon forming exciplex by excitation, undergoes substantial structural relaxation for further stabilization, which takes place with a time constant of several tens to hundreds picoseconds due to the large-amplitude rotaxane-arm relevant motion (Figure 3b).

**Figure 3:**
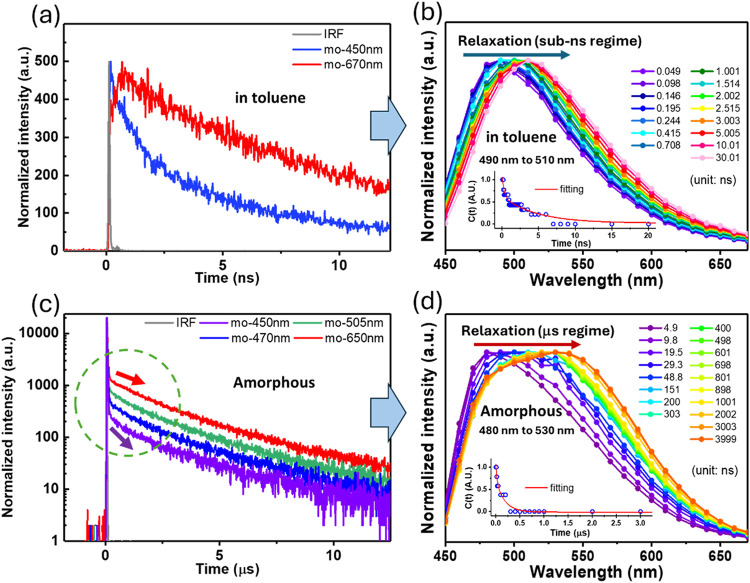
Time-resolved photoluminescence decay of **CT-Rotaxane** in toluene recorded by the TCSPC technique: (a) decay profiles monitored at 450 and 670 nm and (b) spectral–temporal emission map. Inset shows fitting of Stokes shift correlation function, which determines the structural relaxation time constant of 264 ps (see Figure S14). (c) Time-resolved photoluminescence decay of **CT-Rotaxane** in the solid state recorded by the TCSPC technique. The decay profile monitored at 450–670 nm. Purple and red arrows represent the slopes of the dynamic profiles monitored at 450 and 650 nm, respectively. (d) spectral–temporal emission map extracted from (c). Inset shows fitting of Stokes shift correlation function, which determines the structural relaxation time constant of 177 ns.

To quantify this behavior, we thus applied the Stokes shift correlation function *C*(*t*) (SSCF^27,28^ ) expressed in eq 1

1 $$c\left(t\right)=\frac{\nu \left(t\right)-\nu \left(\infty \right)}{\nu \left(0\right)-\nu \left(\infty \right)}$$

where ν(0), ν­(t), and ν(∞) denote the peak emission frequencies measured at *t* = 0, *t*, and ∞, respectively. Equation 1 is commonly used to describe solvent dielectric relaxation dynamics for emissive states undergoing significant dipole moment changes (cf. ground state),^27^ which has also been applied recently to study photoinduced ion migration.^28^ The inset of Figure 3b shows *C*(*t*), and exponential fitting gives a structural relaxation time constant of 264 ps (Figure S14).

To further substantiate the proposed mechanism, we employed the complex **TrMe@Trz-cage** (Scheme 1) as a reference sample (Figure S19c,d), which was previously reported as a strong D­(**TrMe**)/A­(**Trz-cage**) complex,^4^ and acquired the time-resolved spectral evolution. The results revealed in Figure S20a,b, show that the time-resolved emission spectra of **TrMe@Trz-cage** exhibits negligible red shift from 20 ps to μs time scale in toluene. Clearly, in a tightly packed supramolecular assemblies such as **TrMe@Trz-cage**, following Franck–Condon excitation, the extent of structural relaxation is greatly diminished, resulting in a negligible spectral shift in its temporal evolution.

The structural relaxation observed in the solid state requires sufficient void space within the **CT-Rotaxane** environment. This is plausible given the intrinsic flexibility of the cage–thread framework. Supporting evidence comes from the single-crystal structure of **CT-Rotaxane**. To probe the internal free volume, we used the *MoloVol* program^29^ to estimate cavity sizes within the crystallographic unit cell generated in *ChimeraX*.^30^ The total unit cell volume is 12,298.8 Å^3^, within which four **CT-Rotaxane** molecules occupy a combined van der Waals volume of 8,156.2 Å^3^. This corresponds to ∼66.3% occupancy, leaving a substantial void fraction of 33.7%. The solvent-accessible void volume—represented by the small-probe core and shell regions—was calculated to be ∼1496.9 Å^3^ (∼12.2%) and is illustrated in Figure 4a, where arrows mark their locations adjacent to the Cbz-thread. These cavities may serve as buffer space that accommodates motion of the **Cbz-guest** tail. In contrast, the probe-excluded void volume is ∼2645.7 Å^3^ (∼21.5%; ochre regions in Figure 4b), distributed between the **Trz-cage** and the **Cbz-guest**. This void region provides the structural freedom required for relaxation of the Cbz-core moiety in the excited state (see Figures 4a,b, and S5). Assuming the solid powder retains a similar ∼20% void fraction as in the crystal, the observed structural relaxation of **CT-Rotaxane** in the solid state can thus be rationalized.

**Figure 4:**
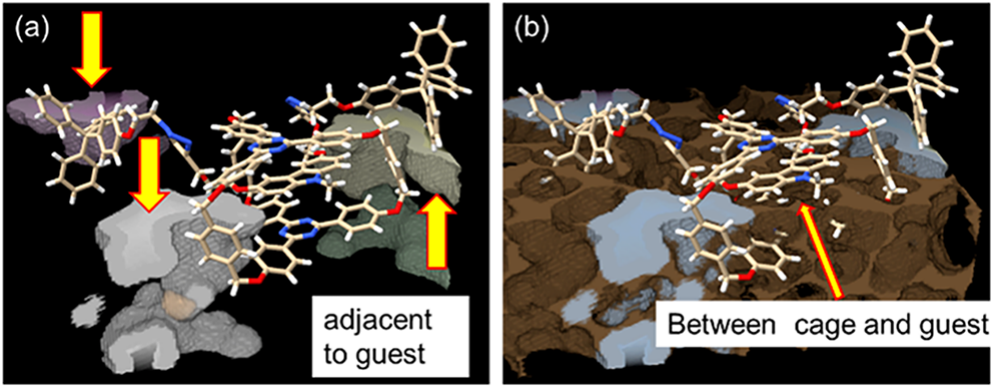
(a) Single-molecule framework with the solvent accessible voids volume highlighted in color rendering. (b) Single-molecule framework with the probe-excluded void volume highlighted in ochre rendering (with the solvent accessible voids volume also shown in gray–blue rendering). The entire unit cell (containing four molecules) and its corresponding voids are shown in Figure S5 of the Supporting Information.

Figures 3d and S13 demonstrate that the solid state (powder) **CT-Rotaxane** also exhibits prominent TADF behavior. However, while the prompt fluorescence lifetime is approximately 18.1 ns, the delayed fluorescence exhibits pronounced differences in curvature within e.g., ∼2 μs, as indicated by the green dashed circle in Figure 3c. The purple and red arrows in Figure 3c represent the slopes of the dynamic profiles monitored at 450 and 650 nm, respectively. The decreased slope toward the red edge of the emission implies that structural relaxation also occurs for **CT-Rotaxane** in the solid state. The dynamic profile (Figure 3c) also infers a gradual red shift of the emission peak from the initial moment to the submicrosecond time scale. This viewpoint is further supported by the spectral temporal evolution of the delayed fluorescence shown in Figure 3d, which, similar to that of **CT-Rotaxane** in solution, reveals the red-shifted peak wavelength at later acquisition time. The difference apparently lies in the time domain of the structure relaxation, which takes as fast as tens to hundreds picoseconds in solution, while as long as several hundred nanoto-micro seconds in solid.

The structure relaxation, unless subject to large interaction barrier, which is not likely to take place in **CT-Rotaxane**, commonly proceeds with continuous motion, accompanied by emission. Therefore, there is no clear-cut regarding decay and rise component (Figure 3c) and it is not feasible to define the relaxation time. However, the emission peak along the structural relaxation, undergoes a continuously red-shifted emission, analogous to the solvent relaxation process. Here, similar correlation function (eq 1) is employed to probe structural relaxation responsible for the observed spectral shift in solid. Accordingly, the plot of *C*(*t*) with respect to acquisition time deduces a relaxation time constant (τ_R_) of 177 ns in solid (see inset of Figures 3d and S15), which is longer than 264 ps (see inset of Figures 3b and S14) in toluene by ∼3 orders of magnitude. The distinction lies in the characteristic time scales: in low-viscosity organic media, structural relaxation of **CT-Rotaxane** proceeds smoothly within tens to hundreds of picoseconds, whereas in the solid state, the intrinsic structural complexity and subtle conformational kinks hinder relaxation, extending the dynamics to the nanosecond or even microsecond regime.

In brief, as far as the host–guest complex adopts a loose configuration (e.g., **CT-Rotaxane**), Franck–Condon vertical excitation drives the system from a less stable geometry toward a more stable structure, provided that the molecular framework affords sufficient degrees of freedom for structural relaxation. This process manifests as a progressively red-shifted spectral–temporal evolution. To further elucidate the relaxation pathway, as elaborated below, we then employ molecular dynamics (MD) simulations to evaluate the probability of structural trajectories, followed by quantum mechanical calculations to characterize the associated potential energy wells.

### Computational Approach

To gain in-depth insight into the correlation between spectral temporal evolution and structural relaxation in **CT-Rotaxane**, we began by performing classical molecular dynamics (MD)^31–33,35^ simulations to identify the most facile modes of motion by characterizing the system’s dynamic behavior in its ground state, followed by its mapping in the excited state in toluene. Details of the methodology are provided in the Supporting Information. To mitigate the formidable computational cost arising from the structural complexity of **CT-Rotaxane**, the two side arms of the guest (compound **5**) were truncated to 1,2-dimethoxyethane groups (see Figure 5a), thereby simplifying the calculations while preserving the carbazole core moiety encapsulated within the **Trz-cage**. The MD simulation results in Figure 5a reveal that the **Cbz-guest’s** motion is largely confined to a two-dimensional plane within the **Trz-cage** cavity, with negligible vertical displacement owing to a stable π–π stacking distance of ≈3.5 Å to the **Trz-cage**—consistent with the crystal structure determined in this work (3.6–3.7 Å). Within the horizontal plane, however, **Cbz-guest** exhibits substantial mobility (see Figure 4b), characterized by a highly anisotropic sliding motion. Specifically, we identified a dominant lateral displacement of ≈6 Å and a secondary, more restricted transverse motion of ≈2 Å. This pronounced anisotropy primarily arises from the slide motion of **Cbz-guest** along a 2D plane (see Figure S22). Although these ground-state simulations do not explicitly model the excited state, they effectively delineate the sterically accessible pathways for structural rearrangement. We therefore hypothesize that, upon photoexcitation, the relaxation process is energetically driven along this dominant, low-energy sliding coordinate.

**Figure 5:**
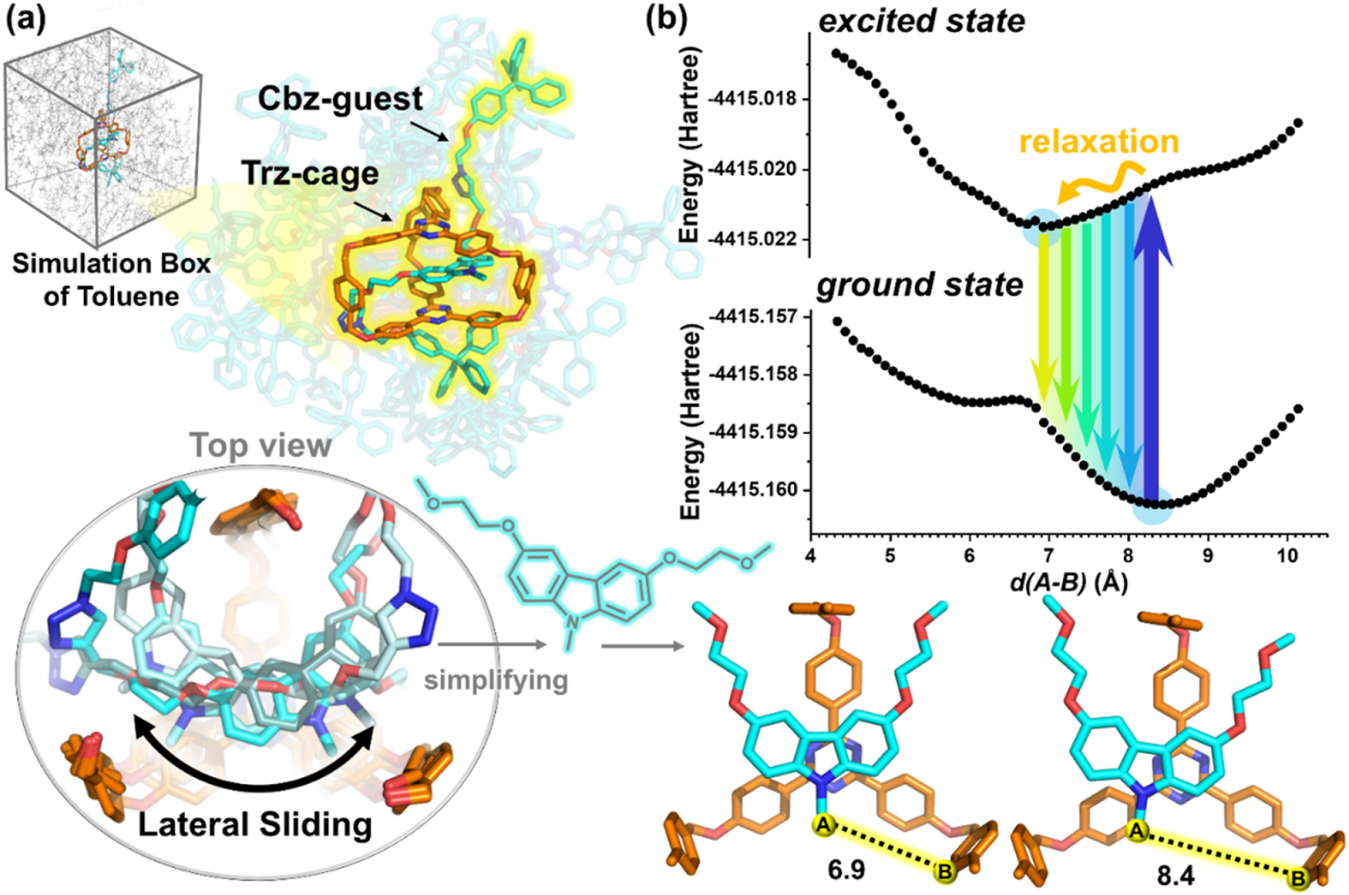
Theoretical investigation of the structural dynamics and relaxation pathway of **CT-Rotaxane**. (a) The snapshots from MD simulations of **CT-Rotaxane** in a toluene solvent box. The top view highlights the confined lateral sliding motion of the **Cbz-guest** within the **Trz-cage**. This observed motion is hypothesized as the primary relaxation coordinate. (b) DFT-calculated PES for the ground and first excited states, plotted as a function of the sliding coordinate. This coordinate is specifically defined by the intermolecular distance *d*(A-B) between the N-methyl carbon of the **Cbz-guest** (A) and a carbon atom on the **Trz-cage** (B), as shown in the schematic. The molecular structures corresponding to the energy minima of both states, highlighted by blue circles on the plots, are displayed at the bottom right. The displacement between the ground and first excited state energy minima validates this structural relaxation pathway. The arrow indicates the relaxation from the Franck–Condon region to the first excited state equilibrium geometry, and the associated energy drop quantitatively accounts for the experimentally observed luminescence redshift.

On this basis, and considering the secondary nature of the transverse motion, our subsequent analysis focused exclusively on the well-defined lateral sliding as the primary structural relaxation coordinate. This key observation from MD simulations led us to hypothesize that lateral sliding constitutes the dominant structural relaxation pathway following photoexcitation. To quantitatively evaluate this hypothesis, we carried out density functional theory (DFT)–based potential energy surface (PES) scans (Gaussian 16 program).^34^ Rather than modeling the full multidimensional sliding motion—which is computationally prohibitive—we introduced a simplified representative coordinate: the intermolecular distance *d*(A–B) between (A) the *N*-methyl carbon of the carbazole moiety in the **Cbz-guest** and (B) a designated carbon atom on the phenyl bridge linking the two triazene units of the **Trz-cage** scaffold (Figure 5b). This *d*(A–B) displacement serves as an effective proxy for tracking the guest’s position during sliding. The PES scan was initiated from the experimentally determined crystal structure to systematically probe the energy landscape along this sliding coordinate.

The small Δ*E*_ST_ of approximately 0.13 eV, as obtained from TD-DFT calculations, suggests an energetically feasible RISC process, consistent with the experimentally obtained Δ*E*_ST_ of 0.084 eV (vide supra), and hence the observed TADF behavior. It should be emphasized that the DFT-based PES analysis presented in Figure 5b is designed to capture the qualitative features of the structural relaxation pathway along the dominant sliding coordinate, rather than to predict absolute excited-state energies. Accordingly, the calculated energy differences are interpreted in a relative sense to elucidate the relaxation mechanism, rather than as quantitatively exact values. As shown in Figure 5b, the ground-state PES plotted as a function of intermolecular distance exhibits a local minimum at approximately 8.5 Å. The potential energy surface of the first excited state was then computed based on the Franck–Condon principle (i.e., vertical excitation), revealing a minimum that is slightly displaced from the ground-state equilibrium geometry. Along the A–B sliding coordinate, the calculated distance *d*(A–B) shifts from 8.5 Å at the Franck–Condon point to 6.9 Å at the relaxed excited-state minimum. This substantial displacement of 1.5 Å quantitatively accounts for the structural relaxation observed experimentally. These results collectively demonstrate that the energy-level distribution of **CT-Rotaxane** is highly sensitive to the intermolecular coupling geometry. The disparity between the ground- and excited-state equilibrium structures is thus responsible for the observed luminescence relaxation. Furthermore, the calculated emission energy difference between *d*(A–B) = 8.4 Å and *d*(A–B) = 6.9 Å is 0.08 eV, in good agreement with the experimentally observed red shift of the emission spectrum from ≈490 to 510 nm (∼0.1 eV). These computational results substantiate the proposed structural relaxation mechanism underlying the bathochromic shift of the emission.

### OLED Performance

We next investigated the OLED properties of the resulting TADF **CT-Rotaxane** as the emissive layer. It is important to note that this study focuses not on achieving record device performance, but rather on comparative evaluation against other TADF complexes, namely **1@Trz-cage** and **TrMe@Trz-cage** (see Scheme 1d). The **1@Trz-cage** complex features a donor moiety structurally analogous to that of **CT-Rotaxane** (cf. compounds **1** and **5** in Scheme 1) but lacks the rotaxane architecture. **TrMe@Trz-cage**, by contrast, represents the first exciplex-based OLED reported with a well-resolved single-crystal X-ray structure,^4^ and likewise adopts a nonrotaxane framework. All three emitters were incorporated into OLEDs using the same device architecture, illustrated in Figure 6a. In brief, **CT-Rotaxane** and **1@Trz-cage** (**or TrMe@Trz-cage**) were each blended with mCP as the host material (weight ratio 6:4) and fabricated into devices with the architecture: ITO (or ITO/PEDOT:PSS)/2PACz/mCP:target/TPBi­(50 nm)/LiF (1 nm)/Al (150 nm). As shown in Figure 6b–d, the device based on **CT-Rotaxane** exhibited a turn-on voltage of 5.4 V and achieved a peak external quantum efficiency (EQE) of 7.23% (Note that as shown in Figure S25, the normalized EL peak wavelength is independent of the voltages applied). At 10.2 V, the device reached a luminance of 500 cd·m^–2^ while maintaining an EQE of 4.95%. The electroluminescence (EL) spectrum displayed a peak emission at 508 nm with a full width at half-maximum (FWHM) of 102 nm. In sharp contrast, the OLED employing **1@Trz-cage** showed significantly inferior performance (Figure S26). The turn-on voltage increased to 9.2 V, and the peak EQE dropped drastically to 0.29%, with a maximum luminance of only ∼30 cd·m^–2^. The EL spectrum exhibited two distinct peaks at 385 and 509 nm. The escape of compound **1** from the **Trz-cage** alters the recombination zone within the emitter bulk, leading to misaligned energy levels and, consequently, short-wavelength emission originating from mCP. This misalignment hinders efficient charge transfer and results in higher turn-on voltages, ultimately giving rise to the overall poor OLED performance.

**Figure 6:**
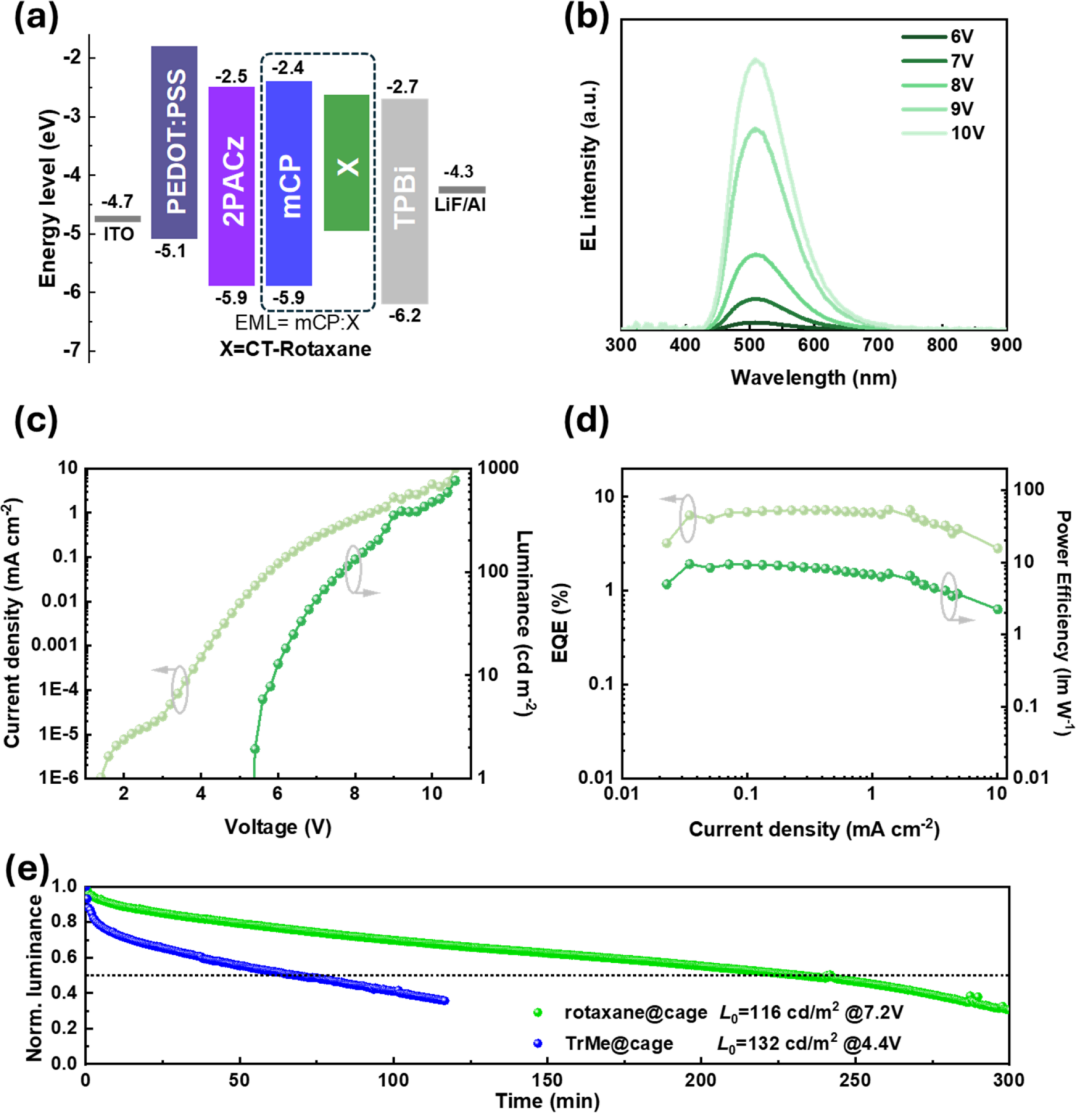
OLED performances based on **CT-Rotaxane** as an emitter. (a) Energy diagram of the OLED device with mCP:**CT-Rotaxane** as the emitting layer. (b) EL spectra of optimized devices at different voltages. (c) Current (light-green line) and luminance (dark-green line) versus voltage curves. (d) EQE (light-green line) and power efficiency (dark-green line) versus current density curves. (e) Comparison of the operational lifetime of OLED devices employing **CT-Rotaxane** (green) and **TrMe@Trz-cage** (blue) as emitters.

Finally, to assess the superior chemical and operational stability of **CT-Rotaxane**, we compared its device performance with that of our previously reported **TrMe@Trz-cage** system.^4^ Although **TrMe@Trz-cage** exhibited higher initial device efficiency, its guest encapsulation relies solely on thermodynamic equilibrium, which inherently limits long-term stability. As shown in Figure 6e, the OLED based on **CT-Rotaxane** demonstrated a markedly enhanced operational lifetime, with an LT_50_ of 231.8 min—over three times longer than that of **TrMe@Trz-cage** (68.1 min). However, despite considerable efforts, TrMe-Rotaxane could not be obtained, likely due to steric hindrance from peripheral functional groups that impeded the threading process. Nonetheless, for **CT-Rotaxane**, the enhanced device stability can be attributed to its mechanically interlocked architecture, which effectively prevents guest dissociation and ensures long-term emission stability

## Conclusion

In summary, we report the synthesis, characterization, and excited-state properties of a rotaxane TADF exciplex, **CT-Rotaxane**, along with its application in OLEDs. This design strategy enables the formation of a kinetically stabilized donor–acceptor complex, allowing detailed investigation of TADF photophysics in solution—where conventional cage-like exciplexes typically suffer from thermal instability. The enhanced stability of **CT-Rotaxane** also facilitates OLED fabrication and significantly prolongs device lifetime. **CT-Rotaxane** exhibits clear TADF behavior, with delayed lifetimes of 4.8 μs in toluene and 4.7 μs in amorphous solid films. Remarkably, the weak D/A interaction induces pronounced excited-state structural relaxation in both media, with time constants of ∼264 ps in toluene and 177 ns in solid, accompanied by time-dependent red-shifted emission spectra. These observations are further supported by combined quantum mechanical and molecular dynamics simulations.

The proof of concept for enhanced exciplex stability is demonstrated through OLED fabrication: **CT-Rotaxane**–based devices exhibit green electroluminescence with a peak external quantum efficiency (EQE) of 7.23% at a luminance of 263 cd·m^–2^—significantly outperforming the reference 1**@Trz-cage** OLEDs (Table 1). Moreover, the operational lifetime of **CT-Rotaxane** OLEDs is four times longer than that of **TrMe@Trz-cage** devices. Collectively, these results highlight mechanically interlocked TADF exciplexes as a promising and practical platform for advancing high-performance OLED technologies.

**Table 1:** Summary of OLED Device Performance Incorporating mCP:**CT-Rotaxane** and **1@Trz-cage** as the Emitting Layer^e^,^f^

OLED	*V*_on_ [V]^a^	EQE_max_ ^b^/PE_max_ ^c^ [%,lm W]	at 500 cd m^–2^ [%, lm W^1–^, V]	EL peak^d^ [nm]	CIE [*x*,*y*]
mCP:**CT-Rotaxane** (6:4 by weight)	5.4	7.23/8.15	4.95/4.05/10.2	508	0.26, 0.48
mCP:**1@Trz-cage** (6:4 by weight)	9.2	0.29/0.12	N/A	385, 509	0.27, 0.45

a *V*_on_, turn-on voltage.

b EQE_max_, maximum external quantum efficiency.

c PE_max_, maximum power efficiency.

d EL peak, electroluminescence peak.

e FWHM, full-width at half-maximum.

f Calculated at 4 V.

## Supplementary Material



## Notes

The experimental section includes reagents and materials, detailed measurements, computational details, crystal preparation, device fabrication, and characterization. ^1^H NMR spectra,^13^C NMR spectra, and the mass spectra for all compounds, and additional reference (PDF)
