# Supplementary material for: Interlocked Rotaxane Enables TADF with Distinct Excited-State Structural Relaxation
Source: J Am Chem Soc. 2026 Jan 29;148(5):5409–18. doi: 10.1021/jacs.5c19031 (PMC12903867; doi:10.1021/jacs.5c19031)
Supplement: Supplementary file 1 [file ja5c19031_si_001.pdf]

Supporting Information *for*

Interlocked Rotaxane Enables TADF with Distinct Excited-State Structural Relaxation

Chuan-Jing Lin[1#], Kai-Hsin Chang [1#], Chun-Yen Lin[1#], Kuan-Hsuan Su[2#], Chieh-Ming Hung,[1#] Yi-Hung Liu[1], Orion Shih[2*], Ken-Tsung Wong[1,3*] and Pi-Tai Chou[1*]

[1] Department of Chemistry, National Taiwan University, Taipei 106319, Taiwan

[2] National Synchrotron Radiation Research Center (NSRRC), Hsinchu 300092, Taiwan

[3] Institute of Atomic and Molecular Science, Academia Sinica, Taipei 106319, Taiwan

[#] Equal contributions

Table of Contents

1. Experimental Procedures 3
2. Synthesis and Characterization 4
3. Crystallographic Data and Experimetal Methods 11
4. Spectroscopic Data 16
5. Theoretical Calculations 34
6. LED Device Research 36
7. NMR and MS Spectra 38
8. References 46

1. Experimental Procedures

General Information.

All commercial reagent were used as supplied without further purification. Thin-layer chromatography (TLC) was performed on Merck silica gel 60 F254 precoated plates (0.25 mm thickness). Column chromatography was carried out using Merck silica gel 60 (particle size: 0.063–0.200 mm). UV detection was performed using an ACE-glass 7825-34 UV immersion lamp (450 W). All reactions were conducted under an argon atmosphere with magnetic stirring. For moisture-sensitive reactions, glassware was dried under vacuum and flame or oven-dried prior to use, followed by argon backfilling before initiating the reaction. Common solvents were dried as follows: tetrahydrofuran (THF) and *N,N*-dimethylformamide (DMF) were purified using solvent purification systems; dichloromethane (DCM) and toluene were dried over phosphorus pentoxide (P_2O_5) and distilled prior to use.

Nuclear Magnetic Resonance (NMR) Spectroscopy.

^{1}H and ^{13}C NMR spectra were recorded on Agilent MERCURY 400 MHz and Bruker AVIII-500 MHz NMR spectrometers. Chemical shifts (δ) are reported in parts per million (ppm), using deuterated chloroform ($CDCl_3$) or deuterated dimethyl sulfoxide ($(CD_3)_2SO$) as solvents. Residual solvent peaks were used as internal standards: $CDCl_3$ at δ 7.26 ppm and DMSO-d_6 at δ 2.50 ppm. The splitting patterns are abbreviated as follows: s = singlet, d = doublet, t = triplet, q = quartet, m = multiplet, br = broad. Coupling constants (*J*) are reported in hertz (Hz). The ^{13}C signals were assigned using Distortionless Enhancement by Polarization Transfer (DEPT) to determine the degree of substitution. The classification is as follows: CH_3 corresponds to primary (1°), CH_2 to secondary (2°), CH to tertiary (3°), and quaternary (4°) for non-protonated carbon atoms.

2. Synthesis and Characterization

Synthesis of **9-methyl-9*H*-carbazole-3,6-diol (2)**

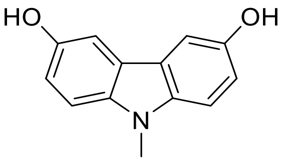

2

Compound **1** (3,6-dimethoxy-9-methyl-9H-carbazole) (1.5 g, 1 eq.) and pyridinium chloride (11.4 g, 15 eq.) were added to a reaction flask and heated to 160 °C. After stirring overnight, the reaction mixture was quenched with water and extracted with ethyl acetate (3 × 70 mL). The organic layer was dried over Na_2SO_4, filtered, and concentrated. The crude product was purified by column chromatography (EtOAc/Hexanes = 1/1), affording compound **2** as a white solid (785 mg, 59%) after solvent removal. ^1H NMR (400 MHz, d_6-DMSO) δ 8.89 (s, 2H), 7.32 (d, J = 2.4 Hz, 2H), 7.28 (d, J = 8.6 Hz, 2H), 6.90 (dd, J = 8.6, 2.4 Hz, 2H), 3.71 (s, 3H). ^{13}C NMR (101 MHz, d_6-DMSO) δ 150.04, 135.59, 122.25, 114.87, 109.37, 104.95, 29.05. HRMS (m/z, MALDI, $[M]^+$) Calcd. for $C_{13}H_{11}NO_2$ 213.0784, found 213.1682. m.p. = 198–200 °C.

Synthesis of **diethyl 2,2'-((9-methyl-9*H*-carbazole-3,6-diyl)bis(oxy))diacetate (3)**

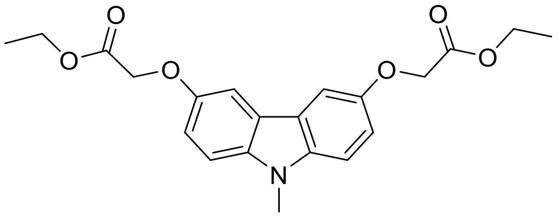

3

Compound **2** (300 mg, 1 eq.) and K_2CO_3 (486 mg, 2.5 eq.) were added to a reaction flask. MeCN (5 mL) was added at room temperature, and the mixture was stirred for 10 minutes. Ethyl 2-bromoacetate (0.4 mL, 2.5 eq.) was then added, and the reaction mixture was heated to 50 °C and stirred for 3 hours. After completion, the solvent was removed, and the residue was extracted with ethyl acetate (150 mL), followed by washing with water (30 mL) and brine (30 mL). The organic layer was dried over Na_2SO_4, filtered, and concentrated. The crude product was purified by column chromatography (EtOAc/Hexanes = 3/7), affording compound **3** as a white solid (525.4 mg, 97%) after solvent removal. ^1H NMR (400 MHz, $CDCl_3$) δ 7.52 (d, J = 2.4 Hz, 2H), 7.28 (d, J = 8.8 Hz, 2H), 7.18 (dd, J = 8.8, 2.4 Hz, 2H), 4.73 (s, 4H), 4.30 (q, J = 7.2 Hz, 4H), 3.78 (s, 3H), 1.32 (t, J = 7.2 Hz, 7H). ^{13}C NMR (101 MHz, $CDCl_3$) δ 169.64, 151.76, 137.53, 122.76, 115.99, 109.52, 105.33, 67.24, 61.46, 29.47, 14.39. HRMS (m/z, MALDI, $[M]^+$) Calcd. for $C_{21}H_{23}NO_6$ 385.1520, found 385.2054. m.p. = 113-114 °C.

Synthesis of **2,2'-((9-methyl-9*H*-carbazole-3,6-diyl)bis(oxy))bis(ethan-1-ol) (4)**

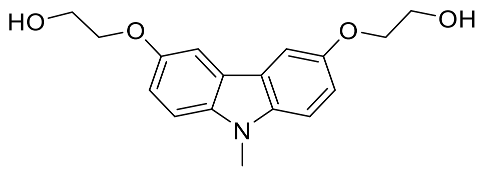

4

Compound **3** (525 mg, 1 eq.) was dissolved in THF (3.4 mL), and the reaction flask was opened at 0 °C for the portion wise addition of $LiAlH_4$ (155 mg, 3 eq.). After hydrogen gas evolution had ceased, the reaction mixture was heated to 45 °C and stirred for 3 hours. The reaction was then cooled back to 0 °C, and quenched by the sequential addition of water (0.5 mL), 10% NaOH(aq.) (0.5 mL), and water (1 mL). The resulting mixture was filtered and washed with hot THF. The combined filtrate was concentrated to afford compound **4** as a white solid (367 mg, 90%). ^{1}H NMR (400 MHz, $CDCl_3$) δ 7.55 (d, *J* = 2.4 Hz, 2H), 7.28 (d, *J* = 8.8 Hz, 2H), 7.14 (dd, *J* = 8.8, 2.4 Hz, 2H), 4.21 (t, *J* = 4.2 Hz, 4H), 4.02 (t, *J* = 4.2 Hz, 4H), 3.81 (s, 3H). ^{13}C NMR (101 MHz, $CDCl_3$) δ 152.36, 137.24, 122.88, 115.86, 109.52, 104.84, 70.69, 61.96, 29.52. HRMS (m/z, MALDI, $[M]^+$) Calcd. for $C_{17}H_{19}NO_4$ 301.1309, found 301.1646. m.p. = 166-168 °C.

Synthesis of **9-methyl-3,6-bis(2-(prop-2-yn-1-yloxy)ethoxy)-9*H*-carbazole (5)**

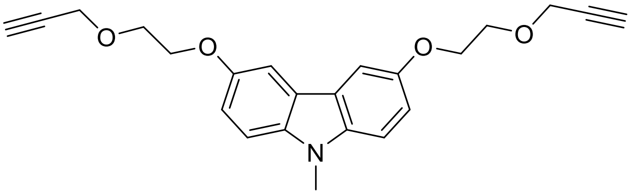

5

Compound **4** (350 mg, 1 eq.) and NaH (60%, 91 mg, 2.5 eq.) were weighed into a reaction flask. Under an argon atmosphere, DMF (3 mL) was added, and the mixture was stirred at room temperature for 10 minutes. Propargyl bromide (0.2 mL, 2.5 eq.) was then added, and the reaction mixture was stirred overnight. After completion, the reaction mixture was concentrated, and the residue was extracted with ethyl acetate (150 mL), followed by washing with water (5 × 30 mL) and brine (30 mL). The organic layer was dried over Na_2SO_4, filtered, and concentrated. The crude product was purified by column chromatography (EtOAc/Hexanes = 3/7), affording compound **5** as a white solid (193 mg, 56%) after solvent removal. ^{1}H NMR (400 MHz, $CDCl_3$) δ 7.54 (d, *J* = 2.4 Hz, 2 H), 7.28 (d, *J* = 8.8 Hz, 2H), 7.14 (dd, *J* = 8.8, 2.4 Hz, 2H), 4.32 (d, *J* = 2.4 Hz, 4H), 4.27 (t, *J* = 4.8 Hz, 4H), 3.95 (t, *J* = 4.8 Hz, 4H), 3.80 (s, 3H), 2.47 (t, *J* = 2.4 Hz, 2 H) ^{13}C NMR (101 MHz, $CDCl_3$) δ 152.40, 137.15, 122.86, 115.99, 109.38, 104.89, 79.76, 77.40, 74.92, 68.68, 58.78, 29.49. HRMS (m/z, MALDI, $[M]^+$) Calcd. for $C_{23}H_{23}NO_4$ 377.1622, found 377.2512. m.p. = 104-106 °C.

Synthesis of **((4-(2-bromoethoxy)phenyl)methanetriyl)tribenzene (6)**

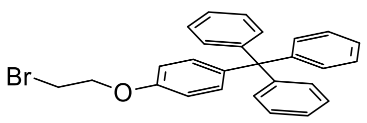

6

4-Tritylphenol (1.0 g, 1 eq.) and K_2CO_3 (622 mg, 1.5 eq.) were added to a reaction flask. Under an argon atmosphere, acetone (33 mL) was added, and the mixture was stirred at room temperature for 10 minutes. 1,2-Dibromoethane (1.6 mL, 6 eq.) was then added, and the reaction mixture was refluxed overnight. The reaction was quenched with water, and the mixture was concentrated. The residue was extracted with CH_2Cl_2 (DCM) (200 mL). The organic layer was dried over Na_2SO_4, filtered, and concentrated. The crude product was purified by column chromatography (DCM/Hexanes = 3/7), affording compound **6** as a white solid (174 mg, 13%) after solvent removal. ^{1}H NMR (400 MHz, $CDCl_3$) δ 7.28–7.18 (m, 15H), 7.16– 7.11 (m, 2H), 6.82–6.79 (m, 2H), 4.30–4.26 (m, 2H), 3.65–3.62 (m, 2H). ^{13}C NMR (101 MHz, $CDCl_3$) δ 156.19, 147.09, 139.98, 132.49, 131.26, 127.63, 126.07, 113.67, 67.91, 64.49, 29.37. HRMS (m/z, MALDI, $[M]^+$) Calcd. for $C_{27}H_{23}{}^{79}BrO$ 442.0927, found 442.1598 Calcd. for $C_{27}H_{23}{}^{81}BrO$ 444.0910, found 444.1636. m.p. = 172–174 °C.

Synthesis of **((4-(2-azidoethoxy)phenyl)methanetriyl)tribenzene (7)**

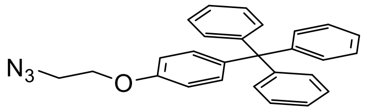

7

Compound **6** (149 mg, 1 eq.) and NaN_3 (218 mg, 10 eq.) were added to a reaction flask. Under an argon atmosphere, DMF (2.6 mL) was added, and the reaction mixture was heated to 80 °C and stirred overnight. The mixture was then extracted with CH_2Cl_2 (80 mL), followed by sequential washing with water (5 × 16 mL) and brine (16 mL). The organic layer was dried over Na_2SO_4, filtered, and concentrated to afford compound **7** as a white solid (136 mg, > 99%). ^{1}H NMR (400 MHz, $CDCl_3$) δ 7.28-7.18 (m, 15H), 7.14 (d, J = 8.5 Hz, 2H), 6.81 (d, J = 8.5 Hz, 2H), 4.13 (t, J = 5.0 Hz, 2H), 3.59 (t, J = 5.0 Hz, 2H). ^{13}C NMR (101 MHz, $CDCl_3$) δ 156.34, 147.10, 139.91, 132.46, 131.27, 127.62, 126.06, 113.53, 66.99, 64.49, 50.37. HRMS (ESI^+): *m/z* Calcd. for $C_{27}H_{23}N_3O[M+Na]^+$: 428.1739, found: 428.1733. m.p. = 156-157 °C.

Synthesis of **9-methyl-3,6-bis(2-((1-(2-(4-tritylphenoxy)ethyl)-1*H*-1,2,3-triazol-4-yl) methoxy)ethoxy)-9*H*-carbazole (Cbz-guest)**

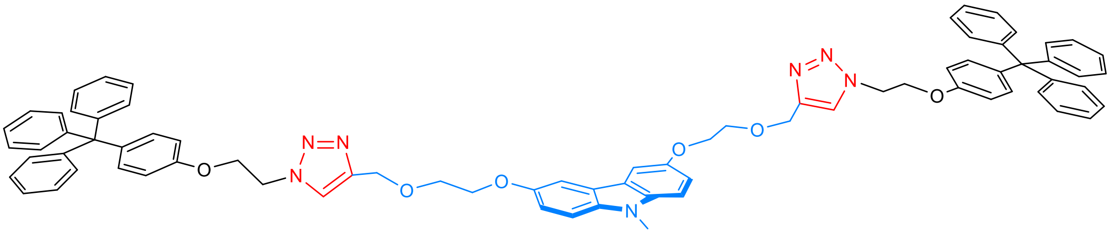

Cbz-guest

Compound **5** (40 mg, 1 eq.) and compound **7** (86 mg, 2 eq.) were added to a reaction flask. Under an argon atmosphere, CH_2Cl_2 (1 mL) was added, followed by the addition of an aqueous *t*-BuOH/water (1:1, 2 mL) solution containing $CuSO_4$ (3 mg, 0.2 eq.) and sodium ascorbate (14 mg, 0.8 eq.). The reaction mixture was stirred at room temperature in the dark for overnight. Upon completion, the mixture was concentrated and extracted with CH_2Cl_2 (60 mL), followed by washing with water (12 mL) and brine (12 mL). The organic layer was dried over Na_2SO_4, filtered, and concentrated. The crude product was purified by column chromatography (MeOH/ CH_2Cl_2 = 2/98), affording Cbz-guest as a white solid (61 mg, 50%) after solvent removal. ^{1}H NMR (400 MHz, $CDCl_3$) δ 7.77 (s, 2H), 7.51 (d, J = 2.4 Hz, 2H), 7.24 – 7.08 (m, 40H), 6.72 (d, J = 8.9 Hz, 4H), 4.79 (s, 4H), 4.70 (t, J = 5.1 Hz, 4H), 4.28 (t, J = 5.1 Hz, 4H), ,4.25 (t, J = 4.8 Hz, 4H), 3.94 (t, J = 4.8 Hz), 3.73 (s, 3H). ^{13}C NMR (101 MHz, $CDCl_3$) δ 155.92, 152.46, 147.02, 145.47, 140.30, 137.06, 132.52, 131.23, 127.66, 126.10, 123.96, 122.83, 116.01, 113.49, 109.37, 104.80, 77.52, 77.40, 77.20, 76.88, 69.41, 68.75, 66.38, 65.03, 64.48, 49.94, 29.46. HRMS (m/z, MALDI, $[M+H]^+$) Calcd. for $C_{77}H_{69}N_7O_6$ 1188.5382, found 1182.5295 (M+1).

Synthesis of **CT-Rotaxane**

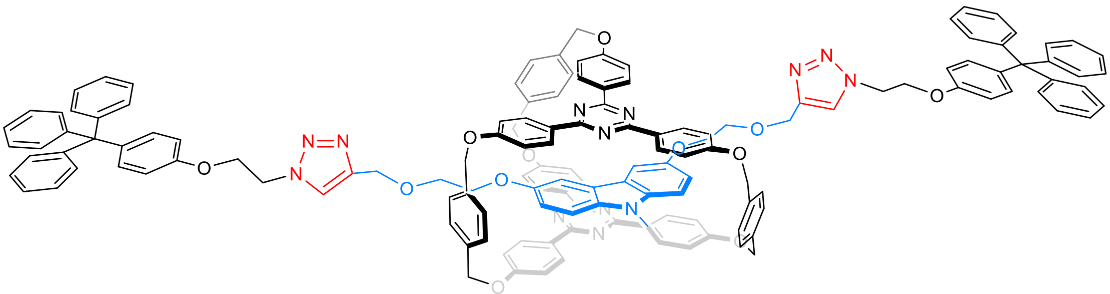

CT-Rotaxane

Compound **5** (100 mg, 1 eq.) and **Trz-cage** (135 mg, 0.5 eq.) were dissolved in CH_2Cl_2. The solvent was then evaporated at 60 °C. Compound **7** (215 mg, 2 eq.) was subsequently added, followed by the addition of CH_2Cl_2 (3 mL) under an argon atmosphere. A solution of $CuSO_4$ (8.5 mg, 0.2 eq.) and sodium ascorbate (37 mg, 0.8 eq.) in water/t-BuOH (1:1, 5.3 mL, for $CuSO_4$) was then added. The reaction mixture was stirred overnight at room temperature in the dark. Upon completion, the mixture was concentrated and extracted with CH_2Cl_2 (300 mL) and water (60 mL), followed by extraction with brine (60 mL). The organic layer was dried over Na_2SO_4, filtered, and concentrated. The crude product was purified by column chromatography (from CH_2Cl_2 to EtOAc/CH_2Cl_2 = 3/7). After solvent removal, the product was further precipitated from CH_2Cl_2/pentane to afford **CT-Rotaxane** as a yellow-green solid (48 mg, 8%). ^{1}H NMR (500 MHz, $CDCl_3$) δ 7.98 (s, 2H), 7.77 (d, J = 8.0 Hz, 8H), 7.72 (d, J = 9.0 Hz, 4H),

7.47 (s, 8H), 7.44 (s, 4H), 7.22-7.12 (m, 32H), 7.07-7.05 (m, 4H), 6.81-6.78 (m, 12H), 6.72-6.69 (m, 4H), 6.01 (dd, *J* = 8.8, 2.3 Hz, 2H), 5.68 (d, *J* = 2.3 Hz, 2H), 5.35-5.25 (m, 12H), 5.10 (d, *J* = 8.8 Hz, 2H), 4.96 (s, 4H), 4.75 (t, *J* = 5.0 Hz, 4H), 4.31 (t, *J* = 5.0 Hz, 4H), 3.93 (t, *J* = 5.0 Hz, 4H), 3.32 (t, *J* = 5.0 Hz, 4H). ^{13}C NMR (126 MHz, $CDCl_3$) δ 169.00 (C), 168.92 (C), 160.16 (C), 160.13 (C), 155.88 (C), 150.75(C), 146.99 (C), 145.72 (C), 140.33 (C), 137.18 (C), 135.01 (C), 132.50 (CH), 131.20 (CH), 130.47 (CH), 130.41 (CH), 129.38 (C), 129.23 (C), 127.72 (CH), 127.64 (CH), 126.07 (CH), 124.05 (CH), 121.30 (C), 115.05 (CH), 114.60 (CH), 114.20 (CH), 113.48 (CH), 108.71 (CH), 101.75 (CH), 77.41 (CH), 69.67 (CH_2), 68.29 (CH_2), 68.09 (CH_2), 66.95 (CH_2), 66.39 (CH_2), 65.12 (CH_2), 64.45 (C), 50.03 (CH_2), 26.49 (CH_3). HRMS (m/z, MALDI, $[M+H]^+$) Calcd. for $C_{143}H_{117}N_{13}O_{12}$ 2209.9049, found 2209.9465.

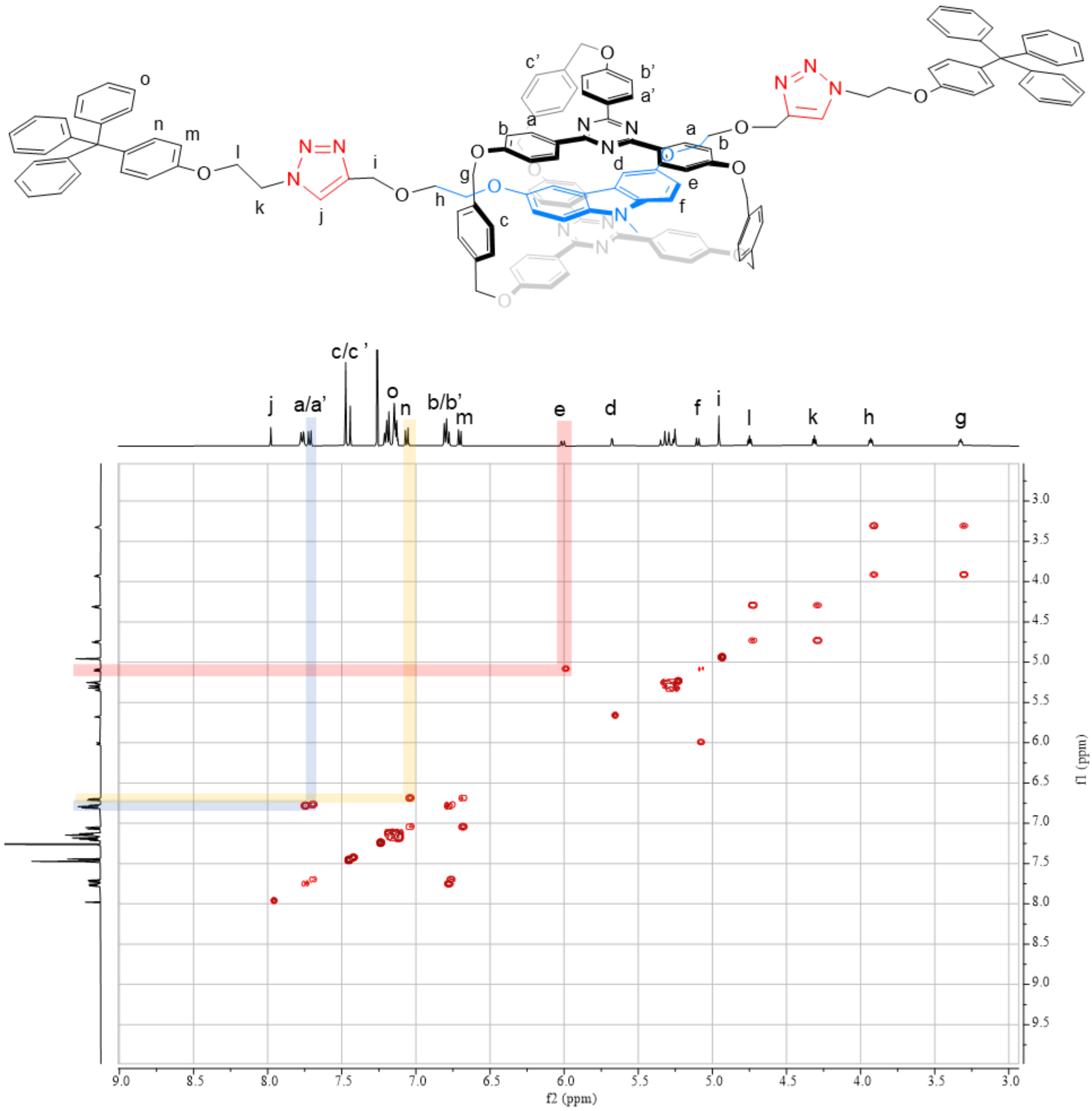

Figure S1. 2D ^{1}H–^{1}H COSY spectrum of **CT-Rotaxane** in $CDCl_3$

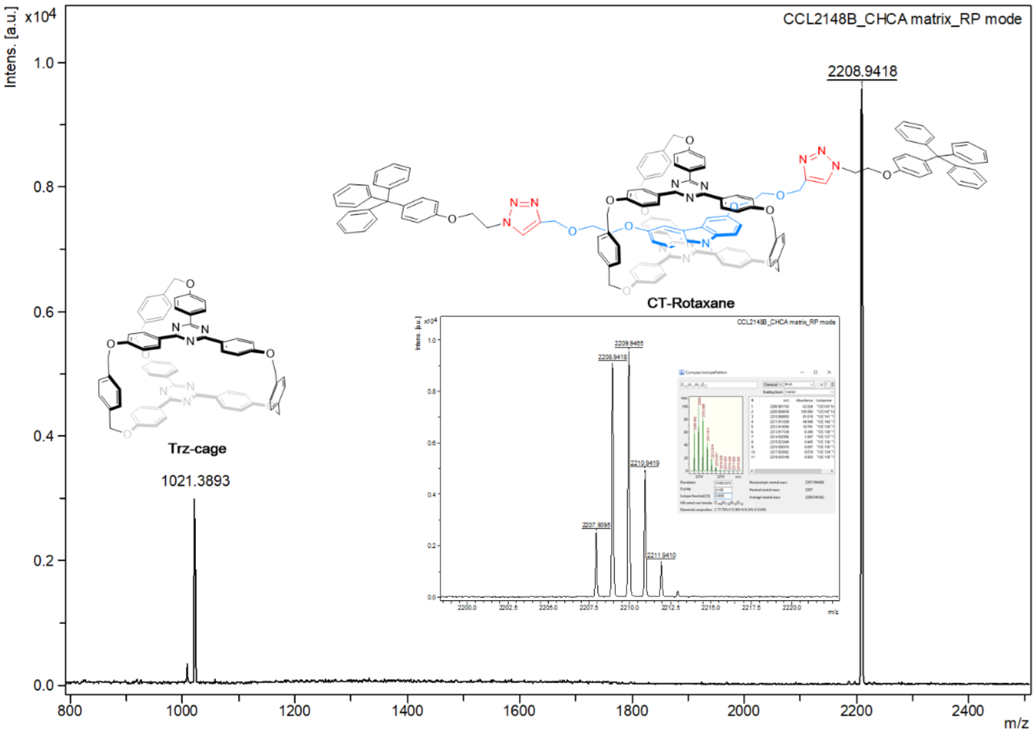

Figure S2. MALDI-TOF Mass Spectrum of **CT-Rotaxane**.

3. Crystallographic Data

Table S1. Summary of crystal structure data.

	**CT-Rotaxane**	**1@Trz-cage**
Empirical formula	$C_{143}H_{116}N_{13}O_{12}$	$C_{82}H_{64}Cl_2N_7O_8$
Formula weight	2208.48	1346.30
T (K) λ (Å)	100(2), 1.54178	100(2), 1.54178
Crystal system Space group	$P2_1/c$	$P2_1/c$
a (Å)	10.2873(4)	25.2967(5)
b (Å)	47.9252(19)	13.3157(2)
c (Å)	24.9459(8)	20.9846(4)
α (°)	90	90
β (°)	90.209(2)	113.2378(7)
γ (°)	90	90
V ($Å^3$), Z	12298.8(8), 4	6495.1(2), 4
R1, wR2 [I>2σ(I)]	0.1320, 0.2816	R1 = 0.0426, wR2 = 0.1095
R1, wR2 (all data)	0.2460, 0.3197	R1 = 0.0492, wR2 = 0.1146
Goodness-of-fit on F^2	1.308	1.017
Largest diff. peak, hole (e $Å^{-3}$)	0.981 and -0.830	0.742 and -0.769

Crystal Structure Determination

Single crystal X-ray diffraction data were collected on Bruker AXS D8 Venture, an IμS microfocus X-ray tube, a Photon-III C28 area detector and an Oxford Cryosystems low temperature device. Examination and data collection were performed with Cu Kα radiation (λ = 1.54178 Å) at 100 K. The frames were integrated with the Bruker SAINT software package. Data were corrected for absorption effects using the Multi-Scan method (SADABS). The space groups were assigned using XPREP within the SHELXTL suite of programs and solved using ShelXT and refined using Shelxl2019 using the graphical interface Olex2. The hydrogen atoms were included in calculated positions and refined using a riding mode.

Crystallographic data along with data collection and refinement details were provided in the table below. Crystallographic data of **CT-Rotaxane** and **1@Trz-cage** were deposited at the Cambridge Crystallographic Data Center with deposition numbers CCDC 2495826 and 2495832, respectively.

Due to the solvent loss there is amount of void volume in the lattice. Consequently, the SQUEEZE function of PLATON was employed to remove the contribution of the electron density associated with these highly disordered solvent. The Squeeze procedure corrected for 427 electrons within the solvent accessible voids.

(a)

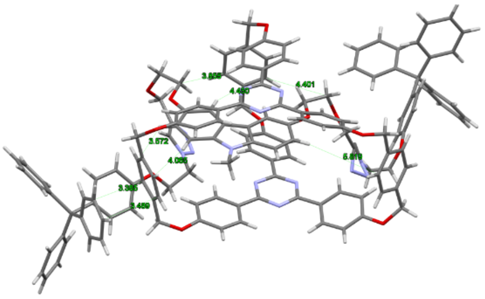

The distance between the thread unit and the pillar components of the **Trz-cage**.

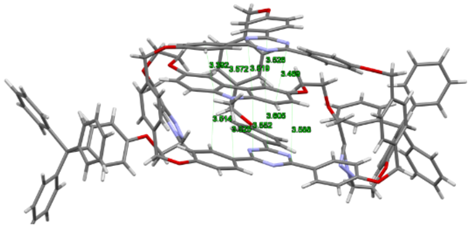

The distance between the central carbazole unit and the two triphenyl triazine lids.

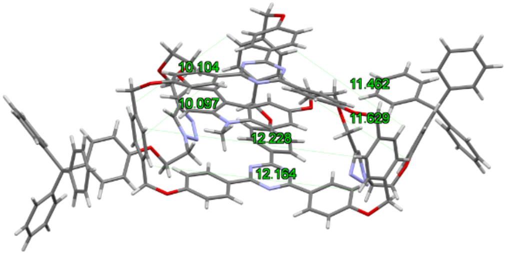

The distance between the openings defined by the two xylylene bridges.

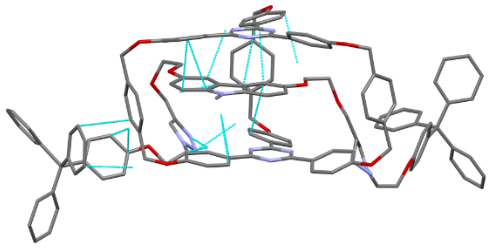

Intramolecular π–π and C–H···π interactions were observed within the CT-Rotaxane structure.

(b)

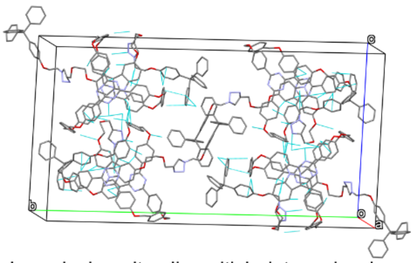

In a single unit cell, multiple intermolecular π–π and C–H··· π interactions are observed between **CT-Rotaxane** molecules.

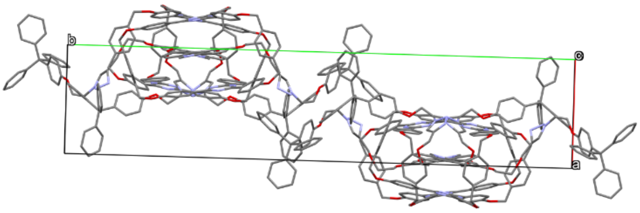

Figure S3. Crystal structure, distance estimations and important interactions listed for (a) **CT-Rotaxane**, and (b) Unit cell of **CT-Rotaxane**; Unit: Å.

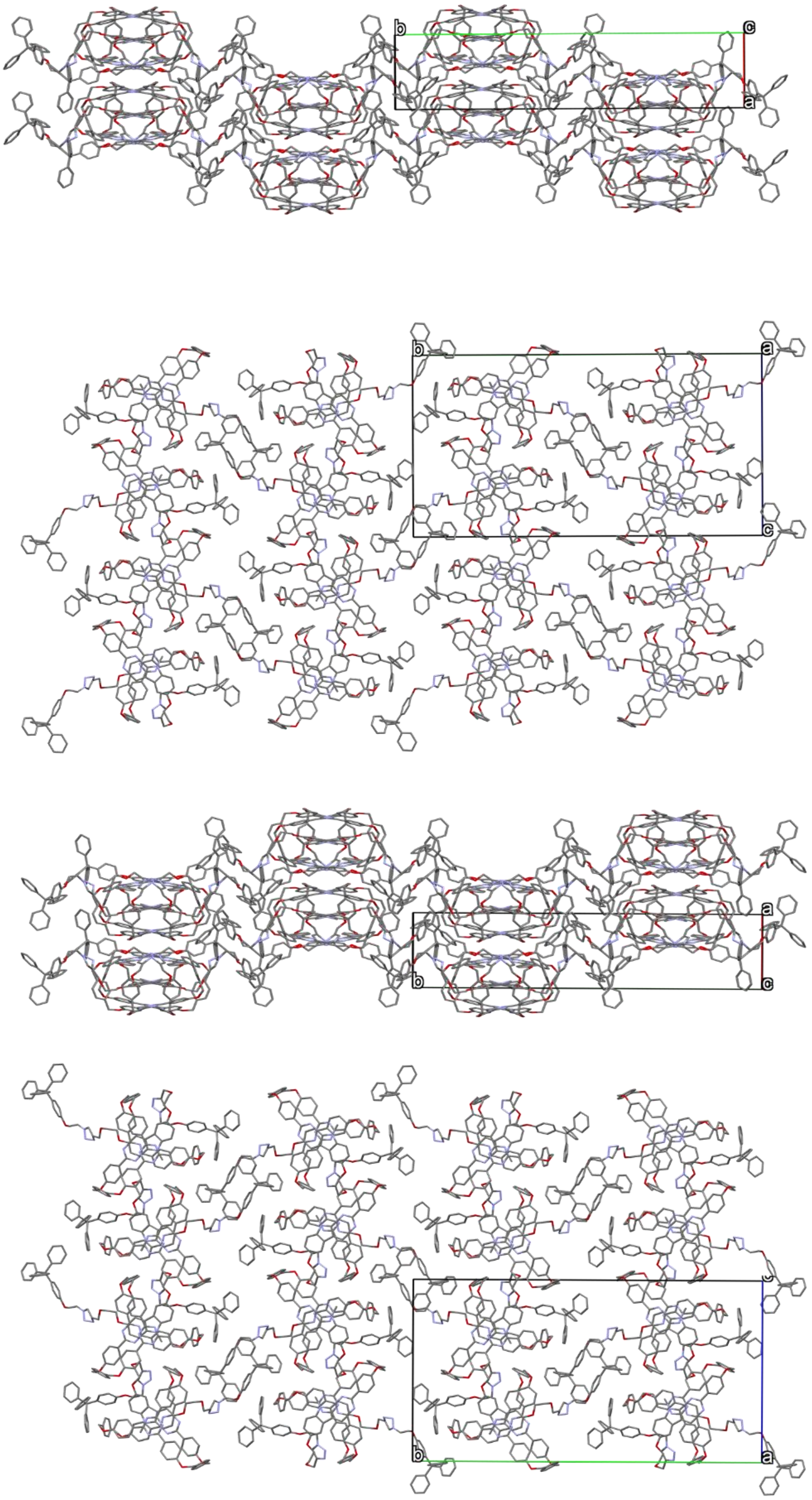

Figure S4. 2×2×2 packing of **CT-Rotaxane**; Unit: Å.

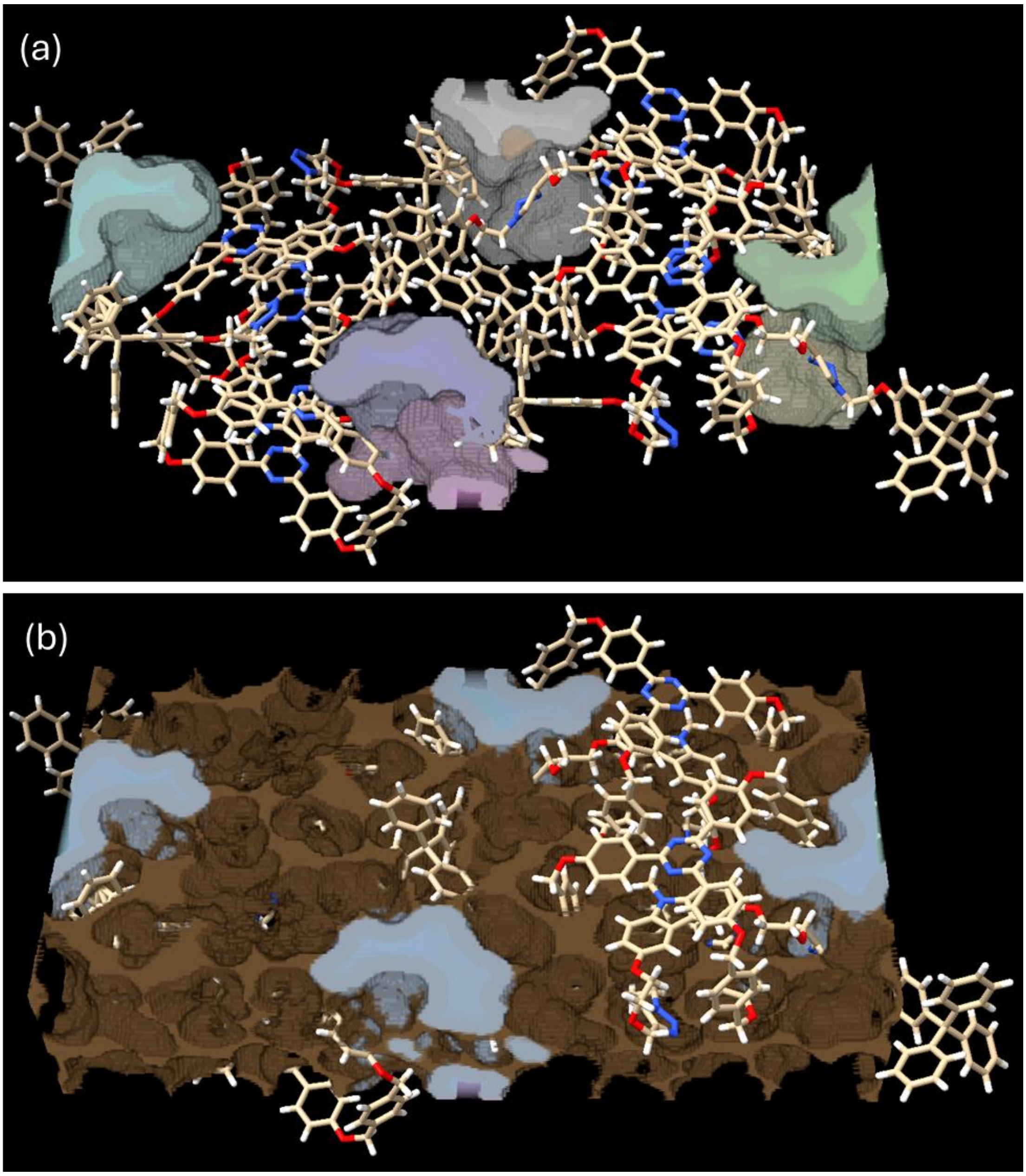

Figure S5. (a) Entire unit cell framework with the solvent accessible void volume highlighted in color rendering. (b) Entire unit cell framework with the probe-excluded void volume highlighted in ochre rendering, with the solvent accessible void volume also shown in gray–blue rendering.

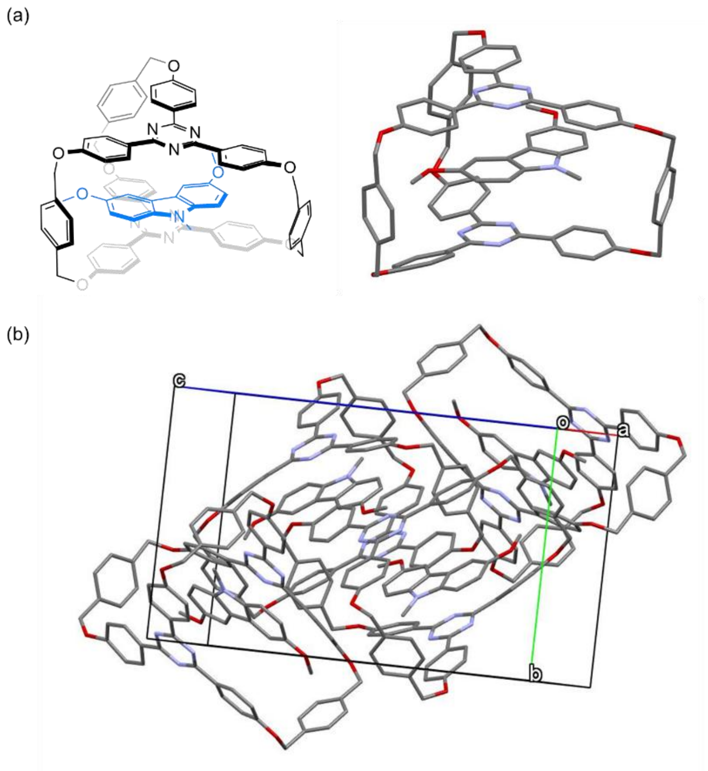

Figure S6. Chemical structure and crystal structure for compound **1@Trz-cage**.

4. Spectroscopic Data

Photophysics Method. The steady-state spectra of the investigated compounds were recorded using an Edinburgh fluorescence spectrometer (FLS-980, Edinburgh Instruments Ltd.) and a Hitachi UV–Vis absorption spectrometer (UH-5700). Spectroscopic-grade solvents (Merck and Acros) were employed to dissolve all solution samples. All solid state samples are amorphous.

Time-resolved measurements in the nanosecond-to-microsecond regime were carried out using the Edinburgh FLS-980 fluorescence spectrometer equipped with a diode laser (Edinburgh Instruments Ltd.). In addition, picosecond-to-nanosecond time-correlated single-photon counting (TCSPC) experiments were performed using a dedicated TCSPC system (Edinburgh Instruments Ltd.) equipped with a microchannel plate photomultiplier tube (MCP-PMT, R3809U-50, Hamamatsu) and coupled with a femtosecond laser (Tsunami, Spectra-Physics) for high-resolution picosecond measurements.

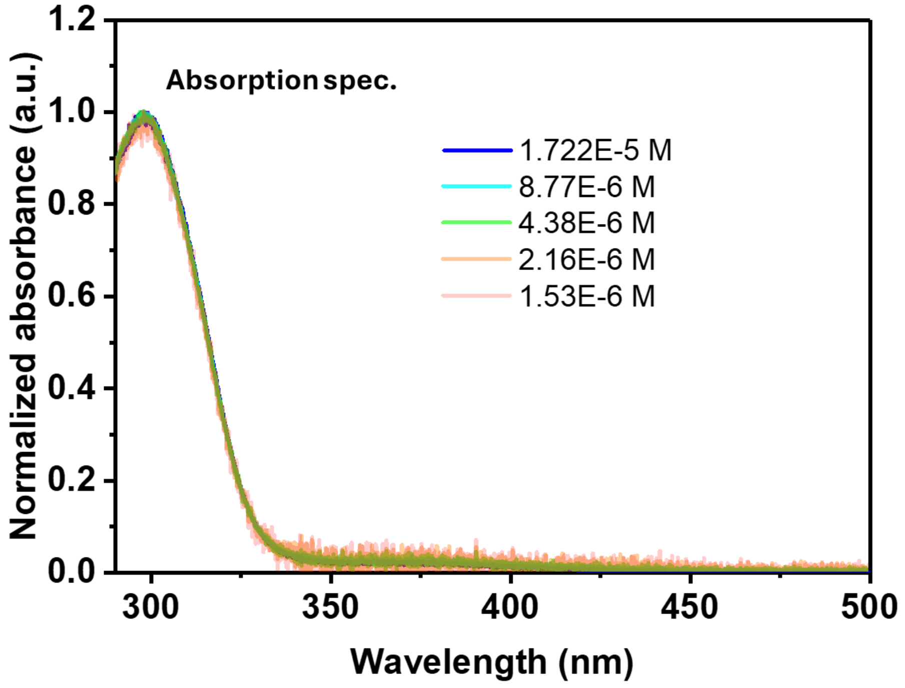

Figure S7. Absorption spectra of **CT-Rotaxane** in toluene at different concentrations, showing no concentration dependence. This indicates that no dimer formation occurs within the investigated concentration range (for spectral experiment, uynless otherwise specified concentration).

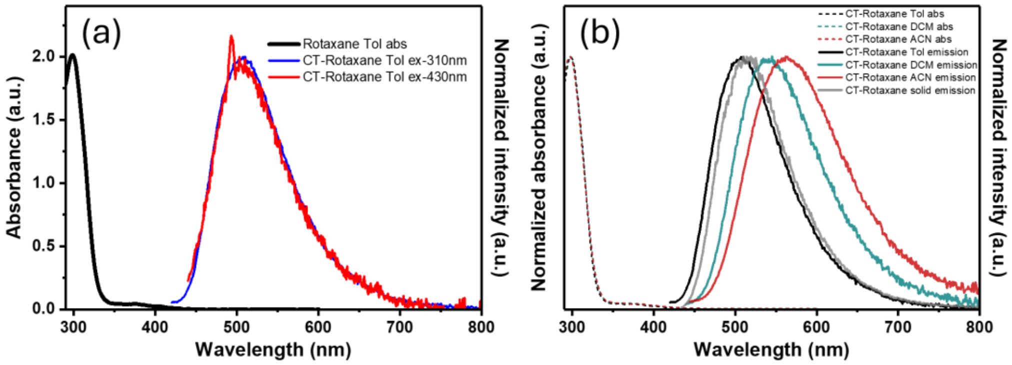

Figure S8. (a) Fluorescence spectra of **CT-Rotaxane** in toluene. (λ_{ex} = 310 nm, 430 nm). (2) The absorption (dashed line) and emission spectra (solid line) of **CT-Rotaxane** in toluene, DCM, ACN, and the solid state.

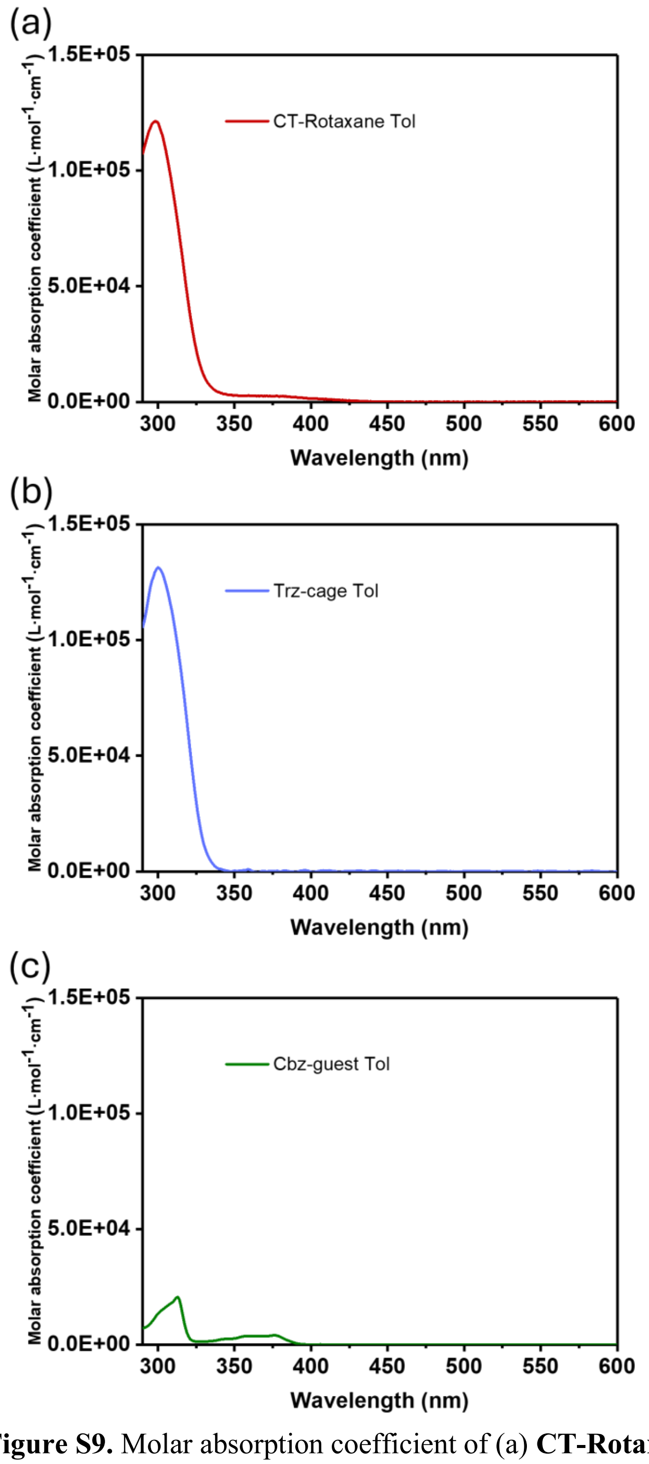

Figure S9. Molar absorption coefficient of (a) **CT-Rotaxane**, (b) **Trz-cage**, (c) **Cbz-guest** in toluene.

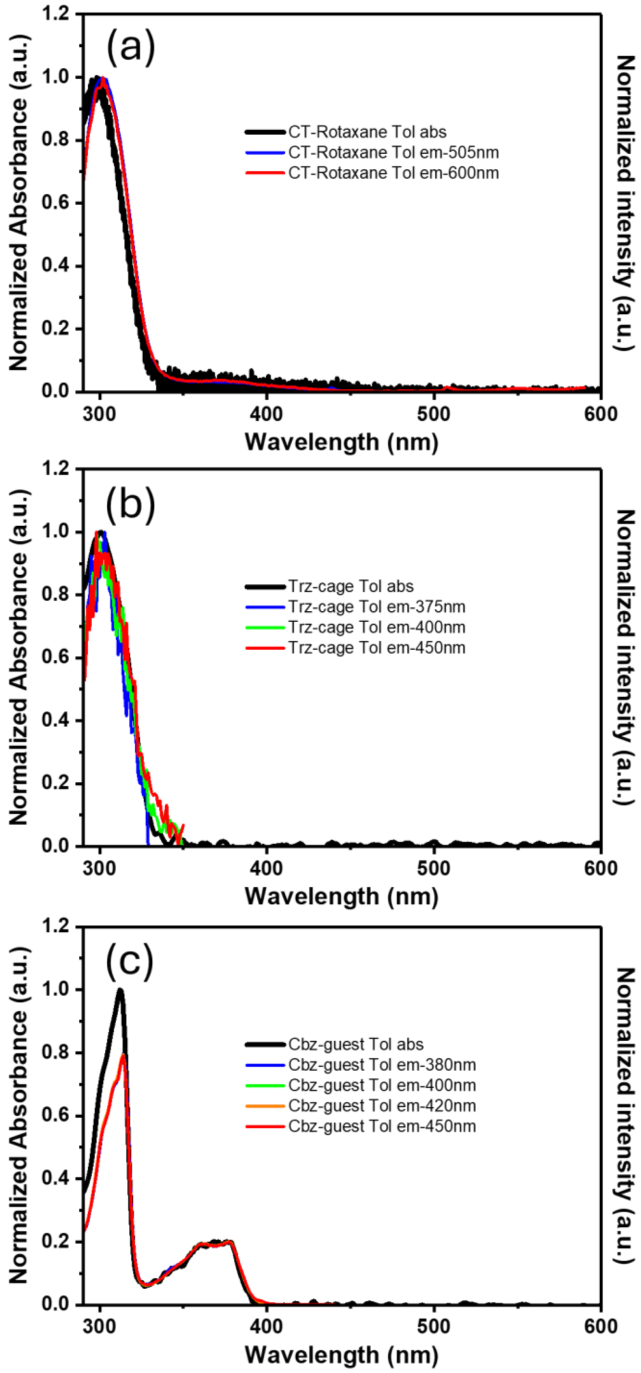

Figure S10. Excitation spectra of **CT-Rotaxane**, **Trz-cage**, and **Cbz-guest**, exhibiting good overlap with their corresponding absorption spectra.

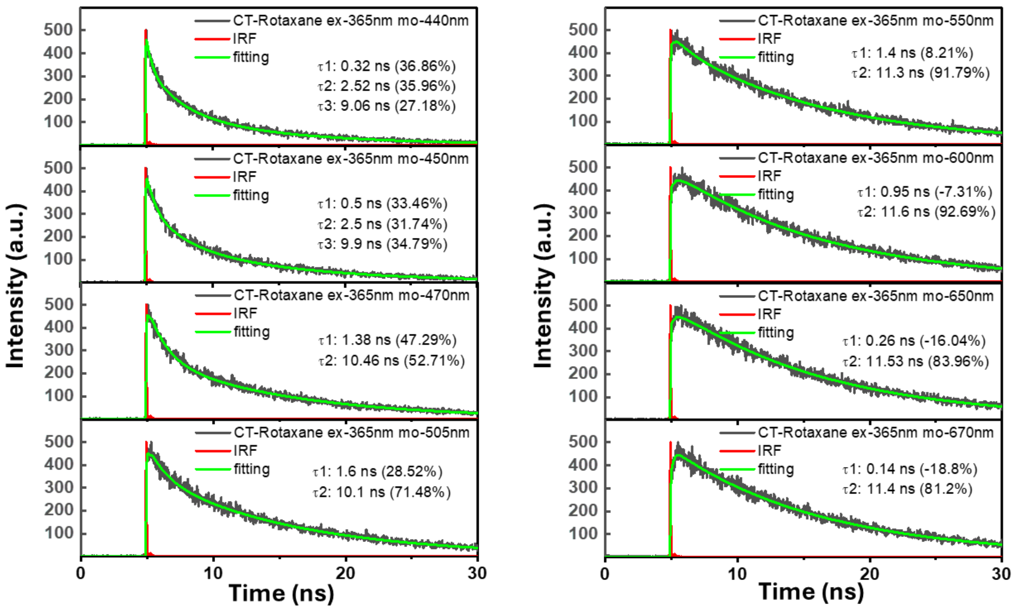

Figure S11. Time-resolved photoluminescence decay profiles of **CT-Rotaxane** in toluene solution. (λ_{ex} = 365 nm).

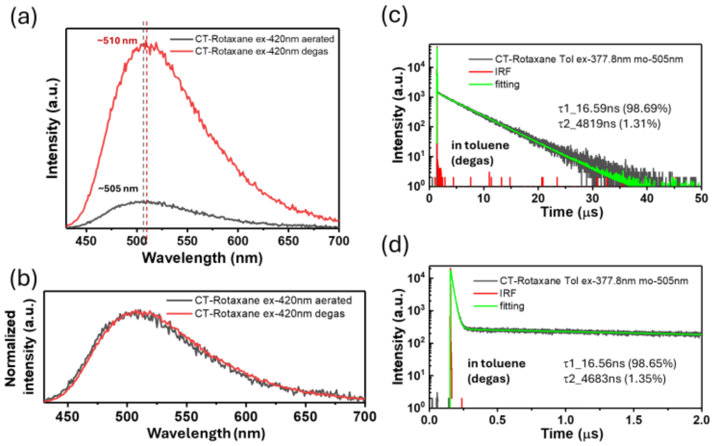

Figure S12. (a) Emission spectra of **CT-Rotaxane** in toluene solution under aerated and degassed conditions. (b) Normalized emission spectra in toluene under aerated and degassed conditions. (c,d) Time-resolved photoluminescence decay profiles of **CT-Rotaxane** in degassed toluene solution (λ_{ex} = 377.8 nm, emission monitored at 505 nm).

Table S2. Calculated rate constants of rotaxane based on the TADF dynamics model. (unit: s^{-1})

Status	k_{ISC}[a]	k_{RISC}[b]	$k^{r}_{singlet}$[c]	$k^{nr}_{singlet}$[d]	$k_{triplet}$[e]	τ_1[f]	τ_2[g]	PLQY[h]
In toluene	4.7699 $\times 10^7$	9.7802 $\times 10^5$	2.7949 $\times 10^6$	8.9965 $\times 10^6$	1.6406 $\times 10^4$	4819 ns	16.59 ns	22.22%

[a] Rate constant of intersystem crossing.
[b] Rate constant of reverse intersystem crossing.
[c] Radiative decay rate constant of fluorescence.
[d] Nonradiative decay rate constant of fluorescence.
[e] Decay rate constant of the triplet state (T_1).
[f] The long-time component obtained from the exponential fitting of the TCSPC dynamic profile.
[g] The short-time component obtained from the exponential fitting of the TCSPC dynamic profile..
[h] Total emissive quantum yield.

The calculation process of Table S2:

For this TADF system, the differential equation is formulated as follows.

$$\frac{d[S_1](t)}{dt} = -k_{ISC}[S_1](t) - k_{singlet}[S_1](t) + k_{RISC}[T_1](t) \quad \text{-eq.(S1)}$$
$$\frac{d[T_1](t)}{dt} = -k_{RISC}[T_1](t) - k_{triplet}[T_1](t) + k_{ISC}[S_1](t) \quad \text{-eq.(S2)}$$
$$[S_1](0) = 1 \quad \text{-eq.(S3)}$$
$$[T_1](0) = 0 \quad \text{-eq.(S4)}$$

Here, $[S_1](t)$ and $[T_1](t)$ represent the time-dependent concentrations of the S_1 and T_1 states, respectively. The differential equations were solved using the Laplace transform, and the resulting analytical solutions are given as follows.

$$[S_1](t) = \frac{[S_1](0)}{\lambda_2-\lambda_1}\{(\lambda_2 - X)e^{-\lambda_1 t} + (X - \lambda_1)e^{-\lambda_2 t}\} \quad \text{-eq.(S5)}$$
$$[T_1](t) = \frac{k_{ISC}[S_1](0)}{\lambda_2-\lambda_1}\{e^{-\lambda_1 t} - e^{-\lambda_2 t}\} \quad \text{-eq.(S6)}$$
$$\lambda_1 = \frac{1}{2}\{(X + Y) - \{(X - Y)^2 + 4k_{ISC}k_{RISC}\}^{\frac{1}{2}}\} \quad \text{-eq.(S7)}$$
$$\lambda_2 = \frac{1}{2}\{(X + Y) + \{(X - Y)^2 + 4k_{ISC}k_{RISC}\}^{\frac{1}{2}}\} \quad \text{-eq.(S8)}$$
$$X = k_{singlet} + k_{ISC} \quad \text{-eq.(S9)}$$
$$Y = k_{triplet} + k_{RISC} \quad \text{-eq.(S10)}$$

By denoting $k^{r}_{singlet}$ and $k^{r}_{triplet}$ as the radiative decay rate constants associated with the S_1 and T_1 states, respectively, the corresponding time-dependent emission intensities can be expressed as $k^{r}_{singlet}[S_1](t)$ and $k^{r}_{triplet}[T_1](t)$, as follows.

Time-dependent emission function:

$$k^{r}_{singlet}[S_1](t) = \frac{k^{r}_{singlet}[S_1](0)}{\lambda_2-\lambda_1}\{(\lambda_2 - X)e^{-\lambda_1 t} + (X - \lambda_1)e^{-\lambda_2 t}\} \quad \text{-eq.(S11)}$$
$$k^{r}_{triplet}[T_1](t) = \frac{k^{r}_{triplet}k_{ISC}[S_1](0)}{\lambda_2-\lambda_1}\{e^{-\lambda_1 t} - e^{-\lambda_2 t}\} \quad \text{-eq.(S12)}$$

The expressions $k^{r}_{single}[S_1](t)$ and $k^{r}_{triplet}[T_1](t)$ can be integrated over time to yield the steady-state fluorescence intensity. Accordingly, the photoluminescence intensity associated with the forward process can be expressed as follows.

Steady-state emission intensity function:

$$Em1 = \int_0^{\infty} k^{r}_{singlet}[S_1](t)dt = \int_0^{\infty} \frac{k^{r}_{singlet}[S_1](0)}{\lambda_2-\lambda_1}\{(\lambda_2 - X)e^{-\lambda_1 t} + (X - \lambda_1)e^{-\lambda_2 t}\}dt \quad \text{-eq.(S13)}$$
$$Em2 = \int_0^{\infty} k^{r}_{triplet}[T_1](t)dt = \int_0^{\infty} \frac{k^{r}_{triplet}k_{ISC}[S_1](0)}{\lambda_2-\lambda_1}\{e^{-\lambda_1 t} - e^{-\lambda_2 t}\}dt \quad \text{-eq.(S14)}$$

The photoluminescence quantum yield of the equilibrium system can be expressed as follows.

$QY_{singlet} = \sum_{n=0}^{\infty} \frac{k_{singlet}^{r}}{k_{singlet}^{r}+k_{singlet}^{nr}+k_{ISC}} \times r^{n} = \frac{k_{singlet}^{r}}{k_{singlet}^{r}+k_{singlet}^{nr}+k_{ISC}} \times \left(\frac{1}{1-r}\right)$ -eq.(S15)

$QY_{triplet} = \sum_{n=0}^{\infty} \frac{k_{triplet}^{r} \times k_{ISC}}{\left(k_{singlet}^{r}+k_{singlet}^{nr}+k_{ISC}\right)\times\left(k_{triplet}^{r}+k_{triplet}^{nr}+k_{RISC}\right)} \times r^{n} = \frac{k_{triplet}^{r} \times k_{ISC}}{\left(k_{singlet}^{r}+k_{singlet}^{nr}+k_{ISC}\right)\times\left(k_{triplet}^{r}+k_{triplet}^{nr}+k_{RISC}\right)} \times \left(\frac{1}{1-r}\right)$ -eq.(S16)

$r = \frac{k_{ISC} \times k_{RISC}}{(k_{singlet}^{r}+k_{singlet}^{nr}+k_{ISC})\times(k_{triplet}^{r}+k_{triplet}^{nr}+k_{RISC})}$ -eq.(S17)

Here, $k_{singlet}^{nr}$ and $k_{triplet}^{nr}$ denote the nonradiative decay rate constants, and $k_{singlet}$ and $k_{triplet}$ are defined as follows.

$k_{singlet} = k_{singlet}^{r} + k_{singlet}^{nr}$ -eq.(S18)

$k_{triplet} = k_{triplet}^{r} + k_{triplet}^{nr}$ -eq.(S19)

From eqs. (S15) and (S16), $k_{singlet}^{r}$ and $k_{triplet}^{r}$ can be expressed as follows.

$k_{singlet}^{r} = QY_{singlet} \times \left(k_{singlet}^{r} + k_{singlet}^{nr} + k_{ISC}\right) \times \left(1 - \frac{k_{ISC} \times k_{RISC}}{\left(k_{singlet}^{r}+k_{singlet}^{nr}+k_{ISC}\right)\times\left(k_{triplet}^{r}+k_{triplet}^{nr}+k_{RISC}\right)}\right) = QY_{singlet} \times \frac{k_{singlet}\times k_{triplet}+k_{ISC}\times k_{triplet}+k_{RISC}\times k_{singlet}}{k_{triplet}+k_{RISC}}$ -eq.(S20)

$k_{triplet}^{r} = QY_{triplet} \times \left(k_{singlet}^{r} + k_{singlet}^{nr} + k_{ISC}\right) \times \left(k_{triplet}^{r} + k_{triplet}^{nr} + k_{RISC}\right) \times \left(1 - \frac{k_{ISC} \times k_{RISC}}{\left(k_{singlet}^{r}+k_{singlet}^{nr}+k_{ISC}\right)\times\left(k_{triplet}^{r}+k_{triplet}^{nr}+k_{RISC}\right)}\right) = QY_{triplet} \times \frac{k_{singlet}\times k_{triplet}+k_{ISC}\times k_{triplet}+k_{RISC}\times k_{singlet}}{k_{ISC}}$ -eq.(S21)

By substituting eqs. (S20) and (S21) into eqs. (S13) and (S14), the following relations are obtained.

$Em1 = QY_{singlet} \times \frac{k_{singlet}\times k_{triplet}+k_{ISC}\times k_{triplet}+k_{RISC}\times k_{singlet}}{k_{triplet}+k_{RISC}} \int_{0}^{\infty} \frac{1}{\lambda_2-\lambda_1}\left\{(\lambda_2 - X)e^{-\lambda_1 t} + (X - \lambda_1)e^{-\lambda_2 t}\right\}dt$ -eq.(S22)

$Em2 = QY_{triplet} \times \frac{k_{singlet}\times k_{triplet}+k_{ISC}\times k_{triplet}+k_{RISC}\times k_{singlet}}{k_{ISC}} \int_{0}^{\infty} \frac{k_{ISC}}{\lambda_2-\lambda_1}\left\{e^{-\lambda_1 t} - e^{-\lambda_2 t}\right\}dt$ -eq.(S23)

Theoretically, the ratio, $\frac{Em1}{Em2}$, should be equal to $\frac{QY_{singlet}}{QY_{triplet}}$, indicating that the residual terms are identical, as shown below.

$\frac{1}{k_{triplet}+k_{RISC}} \int_{0}^{\infty} \frac{1}{\lambda_2-\lambda_1}\left\{(\lambda_2 - X)e^{-\lambda_1 t} + (X - \lambda_1)e^{-\lambda_2 t}\right\}dt = \frac{1}{k_{ISC}} \int_{0}^{\infty} \frac{k_{ISC}}{\lambda_2-\lambda_1}\left\{e^{-\lambda_1 t} - e^{-\lambda_2 t}\right\}dt$ -eq.(S24)

Based on the ratio of pre-exponential factors obtained from the experimental time-resolved profile monitored at 505 nm in the TCSPC measurement, the ratio was determined to be $\frac{0.9869}{0.0131}$ in toluene. The observed decay constants, λ_1 and λ_2, were also measured using TCSPC. In addition, the fluorescence quantum yield was approximated to be close to the total quantum yield, with a value of 22.22%. The relevant equations are presented below.

$$\frac{X-\lambda_1}{\lambda_2-X} \approx \frac{A_2}{A_1} = \frac{0.9869}{0.0131} \quad \text{-eq.(S25)}$$

$$\lambda_1 = \frac{1}{2}\{(X+Y) - \{(X-Y)^2 + 4k_{ISC}k_{RISC}\}^{\frac{1}{2}}\} = (4819 \times 10^{-9})^{-1} \quad \text{(unit: s}^{-1}\text{) -eq.(S26)}$$

$$\lambda_2 = \frac{1}{2}\{(X+Y) + \{(X-Y)^2 + 4k_{ISC}k_{RISC}\}^{\frac{1}{2}}\} = (16.59 \times 10^{-9})^{-1} \quad \text{(unit: s}^{-1}\text{) -eq.(S27)}$$

$$QY_{singlet} = \frac{k_{singlet}^{r}}{k_{singlet}^{r}+k_{singlet}^{nr}+k_{ISC}} \times \left(\frac{1}{1-r}\right) \approx QY_{total} = 22.22\% \quad \text{-eq.(S28)}$$

Where A_1 and A_2 represent the pre-exponential factors (long- and short-time component, respectively) obtained from the exponential fitting of the experimental data. $QY_{singlet}$ denotes the total fluorescence quantum yield, while QY_{total} refers to the overall emissive quantum yield, including both fluorescence and phosphorescence contributions.

Because the ratio of pre-exponential factor, $(X-\lambda_1)/(\lambda_2-X)$, from exponential fitting is very large, it implies the k_{RISC} is very slow. Therefore, we should assumed this equilibrium case as $k_{ISC} + k_{singlet}^{r} + k_{singlet}^{nr} \gg k_{RISC}$, and get the k_{ISC} base on steady-state approximation (the result shown in eq.(S29)).[1]

$$k_{ISC} \approx \lambda_2 \times QY_{ISC} = \lambda_2 \times \frac{QY_{DE}}{QY_{total}} = \lambda_2 \times \frac{\frac{A_1\times(\lambda_2-\lambda_1)}{A_2\times\lambda_1+A_1\times\lambda_2}\times QY_{total}}{QY_{total}} = 4.7699 \times 10^{7} \quad \text{(unit: s}^{-1}\text{)}$$

-eq.(S29) [1]

Where QY_{ISC} is intersystem crossing efficiency, QY_{DE} is photoluminescence efficiencies of delayed emission.

We can solve the system of simultaneous equations given by eqs.(S24)-(S29). The solutions are presented as follows.

$k_{RISC} = 9.7802 \times 10^{5}$ (unit: s^{-1}) -eq.(S30)
$k_{singlet}^{r} = 2.7949 \times 10^{6}$ (unit: s^{-1}) -eq.(S31)
$k_{singlet}^{nr} = 8.9965 \times 10^{6}$ (unit: s^{-1}) -eq.(S32)
$k_{triplet} = 1.6406 \times 10^{4}$ (unit: s^{-1}) -eq.(S33)

In this calculation, we found that k_{RISC} is lower than the S_1 state decay rate ($k_{singlet}^{r}$+$k_{singlet}^{nr}$) in this system. Besides, the slow radiative rate $k_{singlet}^{r}$ arises from the significant HOMO–LUMO spatial separation between the **Cbz-guest** and the **Trz-cage** (see Fig. S23), which is also supported by the corresponding weak oscillator strength (0.0024). Please see *Theoretical Calculations* section of the Supporting Information.

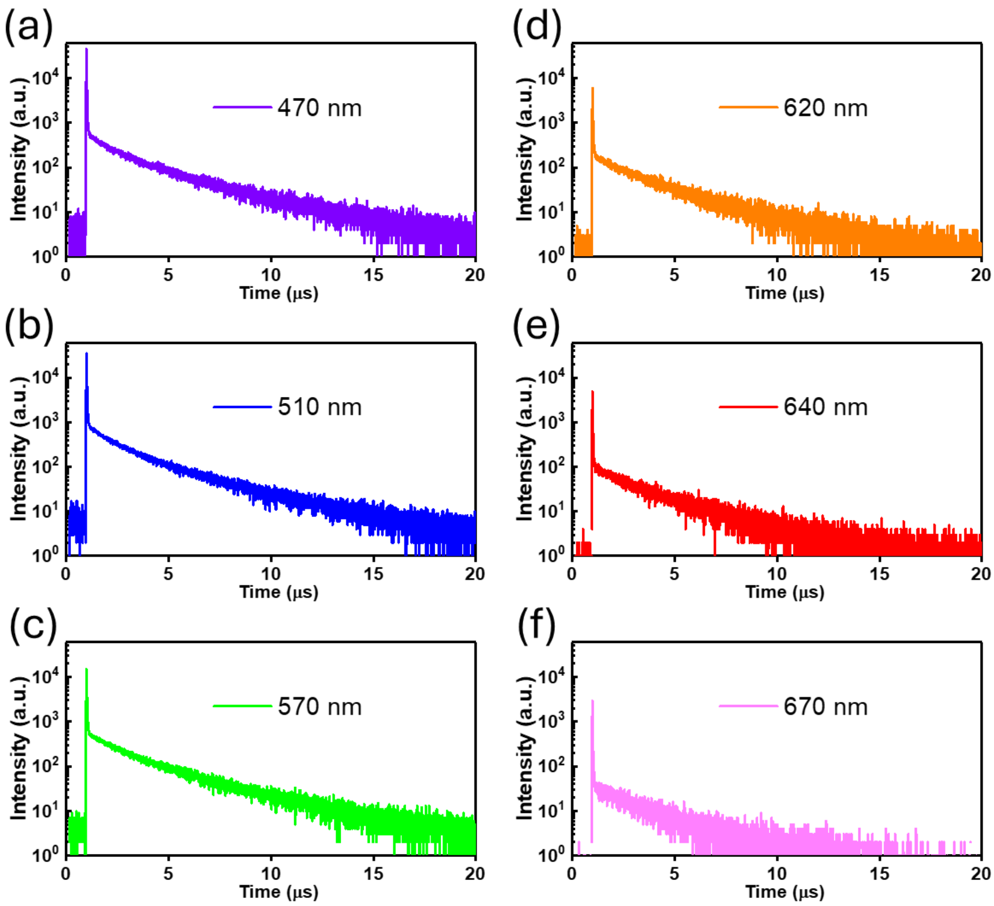

Figure S13. Time-resolved photoluminescence decay profiles of **CT-Rotaxane** (solid state) recorded from 470 to 670 nm (λ_{ex} = 377.8 nm).

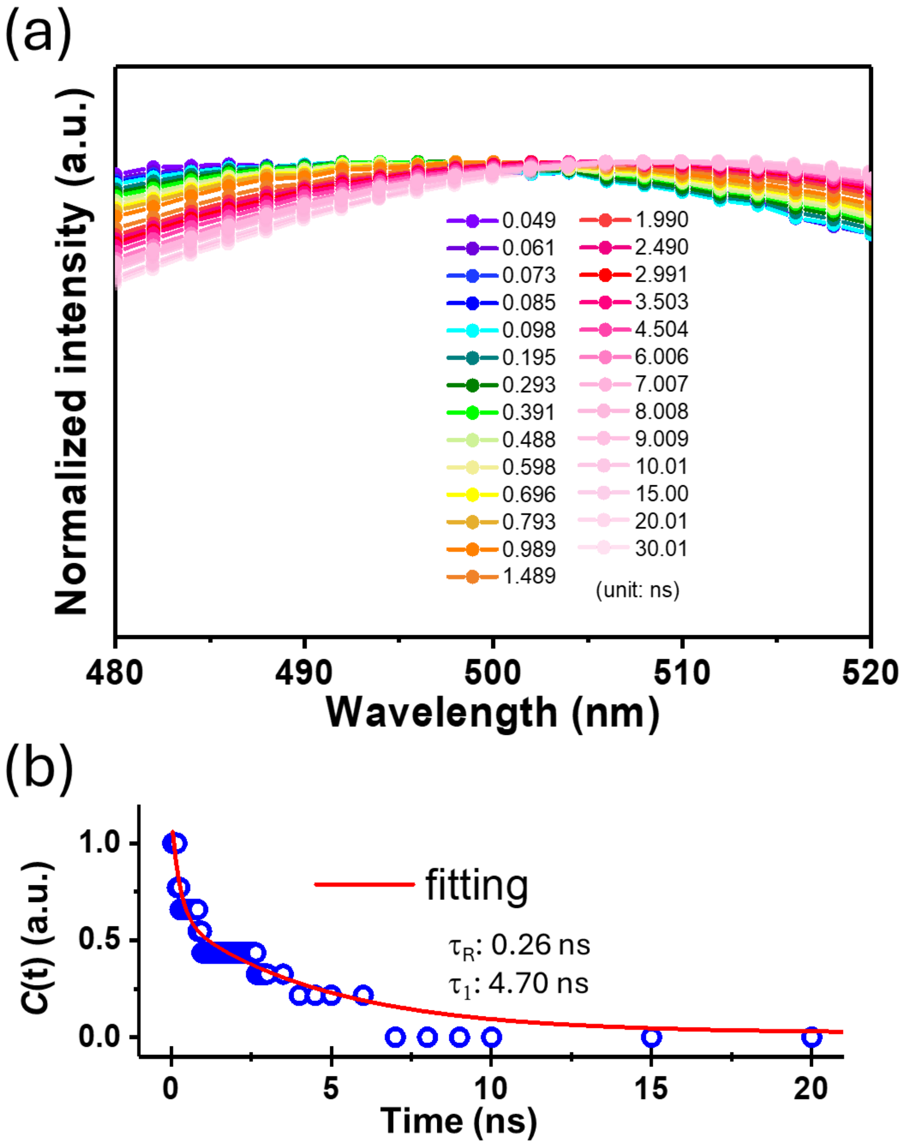

Figure S14. (a) Spectral–temporal emission map of **CT-Rotaxane** in the toluene. (b) Stokes shift correlation function (SSCF) decay profiles of **CT-Rotaxane** in the toluene. τ_R represents the structural relaxation decay time constant (0.26 ns). The fluorescence decay time constant of τ_1 is 4.7 ns. Because structural relaxation is a continuous state-change process, the dynamics cannot be adequately described by a single value of exponential decay. (λ_{ex} = 367.5 nm)

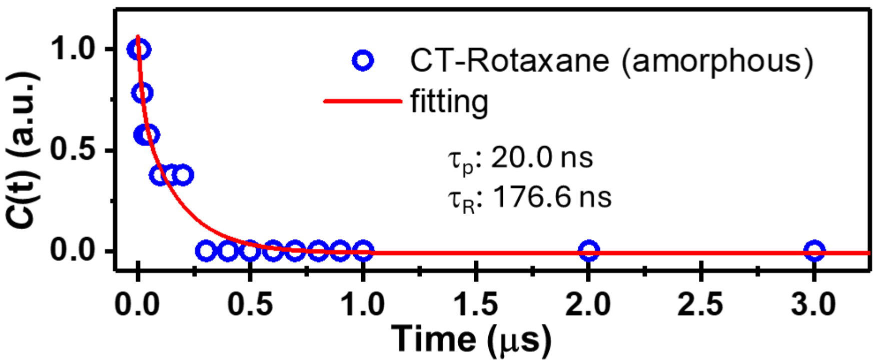

Figure S15. Stokes shift correlation function (SSCF) decay profiles of **CT-Rotaxane** in the solid powder state (λ_{ex} = 377.8 nm). τ_p corresponds to the prompt fluorescence decay (20.0 ns), and τ_R represents the structural relaxation decay time constant (176.6 ns).

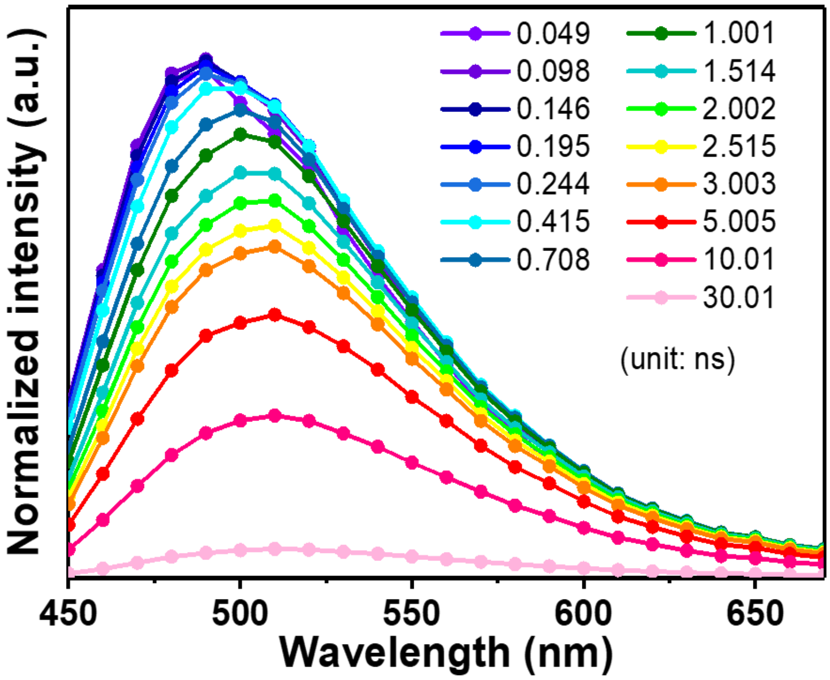

Figure S16. Spectral–temporal emission map (non-normalized) of **CT-Rotaxane** in toluene. (λ_{ex} = 365 nm)

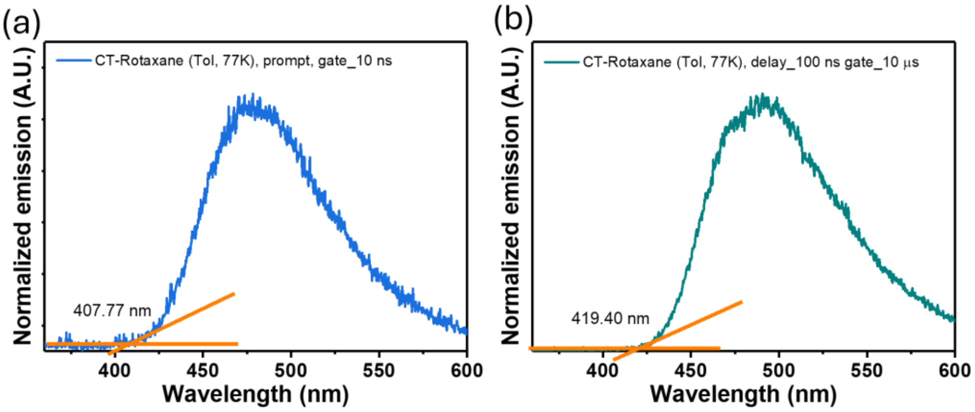

Figure S17. The prompt and delay emission of **CT-Rotaxane** (toluene) in 77K. The ΔE_{ST} is 0.084 eV, which is estimated from the difference between the onset energies of the fluorescence and phosphorescence spectra. (λ_{ex} = 355 nm)

$$\tau(T) = \frac{3+e^{-\frac{\Delta E(S_1-T_1)}{\kappa_B T}}}{3k(T_1)+k(S_1)e^{-\frac{\Delta E(S_1-T_1)}{\kappa_B T}}} \qquad \text{eq. (S34)}$$

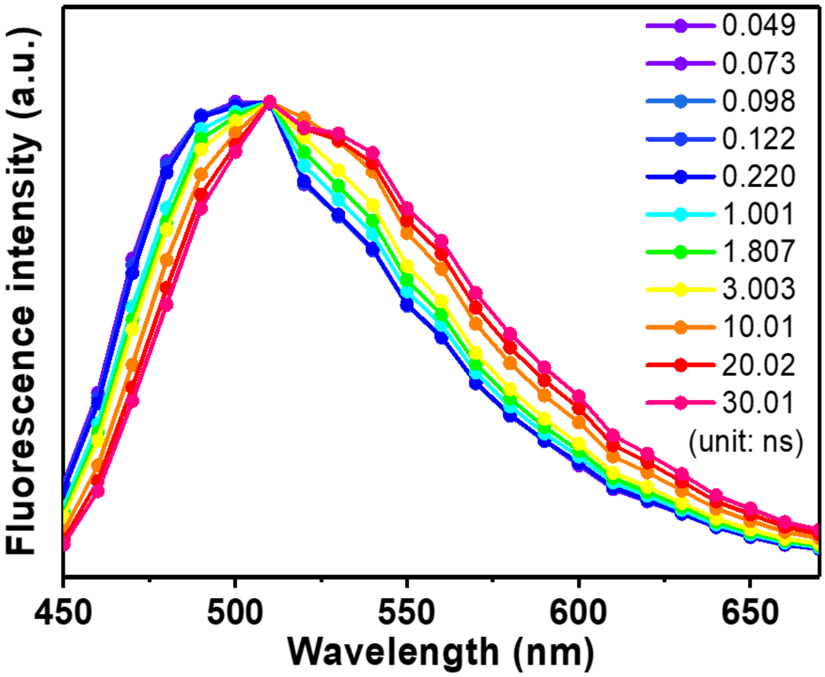

Figure S18. Spectral-temporal evolution of **CT-Rotaxane** in the solid state, measured by picosecond TCSPC (MCP-PMT).

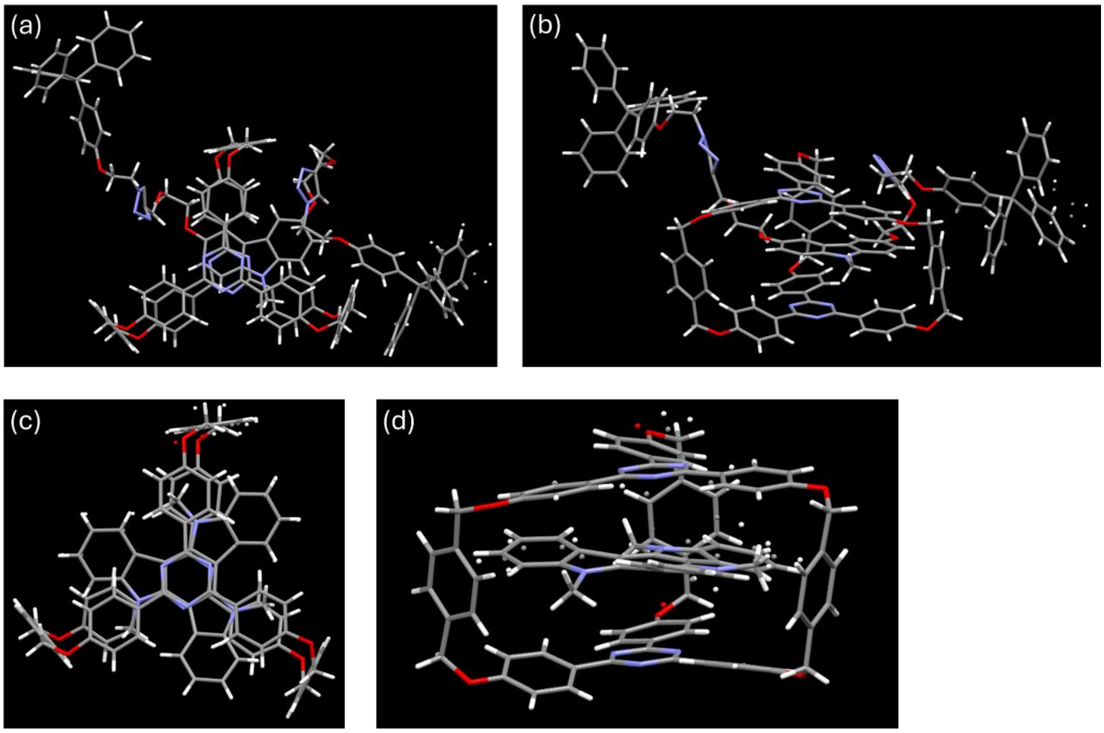

Figure S19. (a) Top and (b) side views of the crystal structure of **CT-Rotaxane**. (c) Top and (d) side views of the crystal structure of **TrMe@Trz-cage** (CCDC 2245433). Because TrMe is larger than carbazole, **CT-Rotaxane** exhibits a larger void fraction within the cage compared to **TrMe@Trz-cage**.

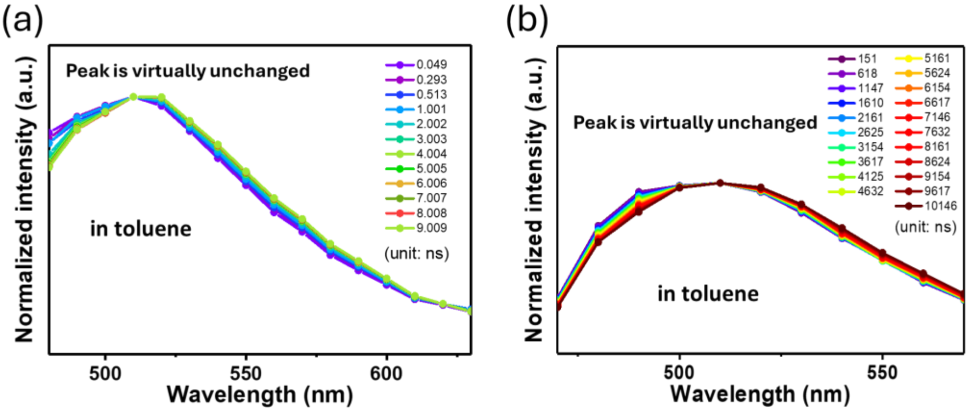

Figure S20. Spectral–temporal evolution of **TrMe@Trz-cage** (toluene) in (a) nanosecond and (b) microsecond regime, measured using picosecon (MCP-PMT) and nanosecond (FLS-980) TCSPC instruments, respectively.

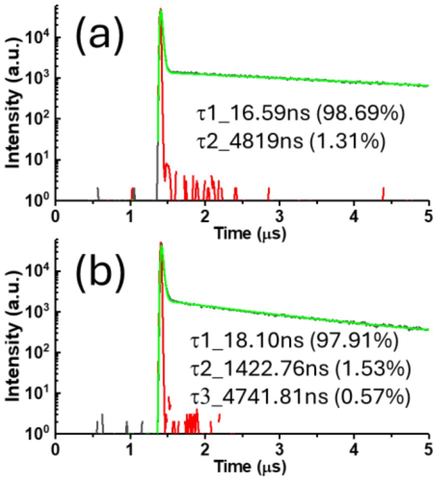

Figure S21. The prompt fluorescence dynamics profiles of **CT-Rotaxane** in toluene (degassed) (a) and solid powder (b). The emission dynamics profile was monitored at 505 nm.

5. Theoretical Calculations

Molecular Dynamics (MD) Simulations. All simulations were conducted using the AMBER 24 software package.[2] The initial structure of the **CT-Rotaxane** is built by the crystal structure determined in this work. The solvated system was constructed using PACKMOL by placing a single rotaxane molecule at the center of a rectangular box and solvating it with 600 explicit toluene molecules, resulting in initial box dimensions of approximately 86 × 45 × 40 $Å^3$.[3] The system was parameterized using the General Amber Force Field (GAFF).[4-5] Partial atomic charges for the rotaxane were derived via the restrained electrostatic potential (RESP) fitting procedure, which was based on quantum mechanical calculations performed at the HF/6-31G* level of theory with Gaussian 16.[6] All simulations were performed with a **1 fs** timestep, where bonds involving hydrogen were constrained by the **SHAKE** algorithm and long-range electrostatics were calculated using the Particle Mesh Ewald (PME) method using a 10.0 Å cutoff. The system was initially minimized through 5,000 steps of steepest descent followed by 5,000 steps of conjugate gradient, with all atoms positionally restrained by a 100 kcal $mol^{-1}Å^{-2}$ force constant. Subsequently, the system was gradually heated from 0 to 300 K over 30 ns within the NVT ensemble, regulated by a Langevin thermostat with a 1.0 ps^{-1} collision frequency. To ensure proper system density, equilibration was conducted at 300 K under the NPT ensemble, where the pressure was maintained at 1 atm using a Berendsen barostat. Finally, a 100 ns production trajectory was generated in the NVT ensemble at 300 K, with system coordinates recorded every 1 ps for subsequent analysis.

Density Functional Theory (DFT) Calculations.

All quantum mechanical calculations were carried out with the Gaussian 16 software package.[6] Given the computational demands of the whole system, the ground-state Potential Energy Surface (PES) was mapped by performing a relaxed scan along a simplified intermolecular coordinate. This coordinate was defined as the distance *d*(A-B) between the N-methyl carbon of the carbazole moiety (A) and a specific carbon atom on the **Trz-cage** scaffold (B). The CAM-B3LYP functional and the 6-31G(d,p) basis set were used for these calculations. Subsequently, vertical excitation energies to the first excited singlet state were computed at each point on the ground singlet state PES using time-dependent DFT (TD-DFT). In accordance with the Franck-Condon principle, these calculations used the same functional and basis set.

Natural Transition Orbital (NTO) analysis was carried out based on the TD-DFT transition density to characterize the electronic nature of the lowest singlet excited state. For the S_1 state, the transition density was decomposed into the corresponding hole (HONTO) and electron (LUNTO) orbitals. The corresponding NTOs were generated and visualized using the Multiwfn program.[7,8]

Additionally, excited-state energy level calculations were performed using both the CAM-B3LYP and ωB97X-D range-separated hybrid functionals in conjunction with the 6-31G basis set. Vertical excitation energies of the S_1 and T_1 states were evaluated to estimate the relative singlet-triplet energy gap (ΔE_{ST}) and to assess the functional dependence of the predicted excited-state energetics. These calculations were intended to provide conceptual and qualitative insight into excited-state energy level alignment and functional trends, rather than quantitative predictions across the full conformational ensemble. Based on the above considerations, the excited-state energy level diagrams shown in Fig. S24 are constructed to provide a qualitative, concept-level representation of the relative alignment of the singlet and triplet excited states. The crystal structure of 1@Trz-cage corresponds to a precursor host-guest complex prior to stopper installation and mechanical interlocking, and thus does not represent a conformer of the fully formed CT-rotaxane. Nevertheless, this structure captures the essential donor–acceptor arrangement and charge-transfer interaction motif relevant to the exciplex-forming unit. Accordingly, the calculated S_1 and T_1 energies should not be interpreted as quantitative predictions for the full conformational ensemble of CT-rotaxane. Despite these limitations, the TD-DFT results consistently indicate a charge-transfer dominated S1 state and a small ΔE_{ST}, supporting the energetic feasibility of reverse intersystem crossing (RISC) in agreement with the experimentally observed TADF behavior.

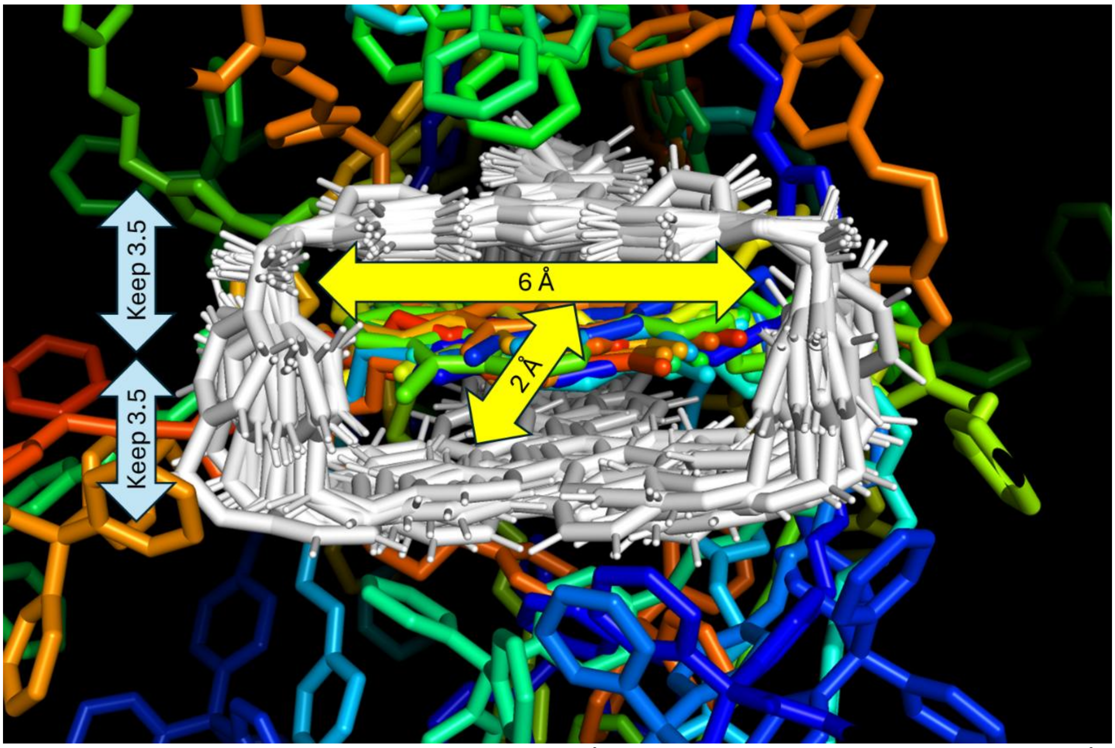

Figure S22. Dominant lateral displacement (~6 Å) and secondary transverse motion (~2 Å) identified for the cage system. The pronounced anisotropy originates mainly from steric hindrance imposed by the bulky trityl-based stopper groups, which restrict molecular movement during displacement.

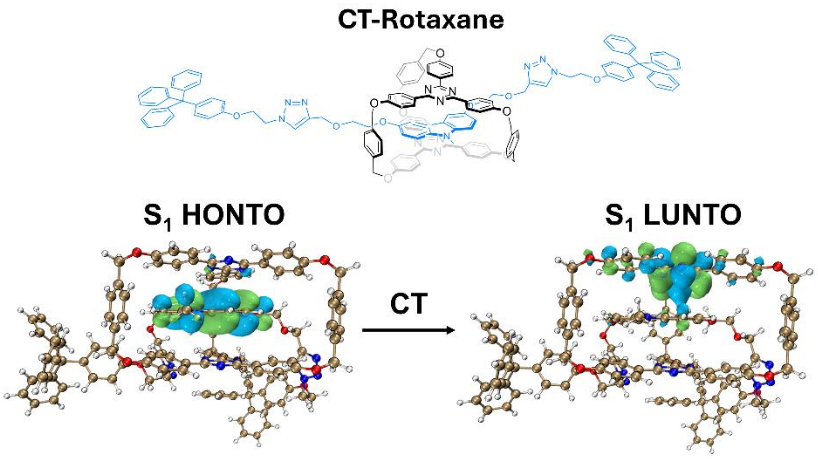

Figure S23. NTO analysis of the S_1 state for **CT-Rotaxane**. The S_1 state is predominantly described by a single transition (97.3% contribution) with a calculated hole-electron centroid distance of 3.27 Å. The HONTO (hole) is primarily distributed over the carbazole-based guest moiety, whereas the LUNTO (electron) is localized on the triazine-containing host cage. This clear spatial separation between the occupied and unoccupied transition orbitals across the interlocked interface confirms the TSCT nature of the lowest singlet excited state in the rotaxane architecture.

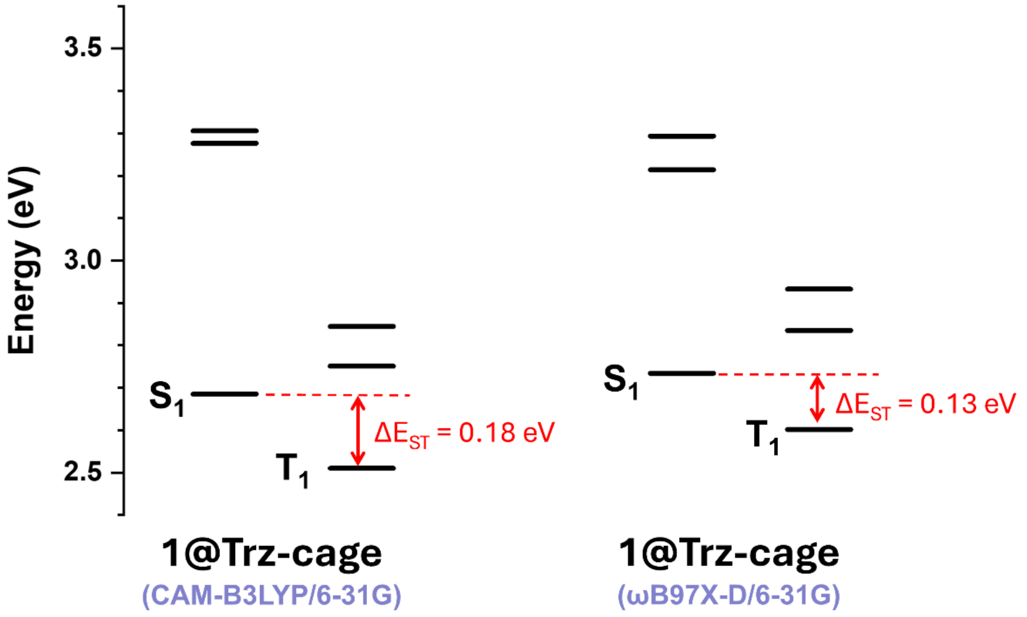

Figure S24. Conceptual excited-state energy diagrams of **1@Trz-cage** calculated by TD-DFT at the indicated levels of theory. Owing to the pronounced conformational flexibility of the **CT-rotaxane** architecture, the crystal structure of **1@Trz-cage** represents only one of many accessible conformers. Consequently, the computed S_1 and T_1 energies should be interpreted in a qualitative rather than quantitative manner. Nevertheless, the S_1 state exhibits pronounced CT character, and the resulting small ΔE_{ST} is consistent with an energetically feasible RISC process, in agreement with the experimentally observed TADF behavior.

6. LED Device Research

Chemicals

PEDOT:PSS, Clevios™ PVP AI 4083, was purchased from Heraeus. (2-(9H-carbazol-9-yl)ethyl)phosphonic acid (2PACz, 99%), 2,2',2"-(1,3,5-Benzinetriyl)-tris(1-phenyl-1-H-benzimidazole) (TPBi, 99%), 1,3-Di(9H-carbazol-9-yl)benzene, N,N′ -Dicarbazolyl-3,5-benzene (mCP, 99%) and Lithium fluoride (LiF, 99.99%) was purchased from Lumtec. Ethanol (EtOH, anhydrous, 99.5%), anisole (anhydrous, 99.5%) and Chlorobenzene (CB, anhydrous, 99.5%) were purchased from Sigma Aldrich.

Device Fabrication

The Compound **1** OLED was fabricated with the device structure of ITO/PEDOT:PSS/2PACz/ mCP:target /TPBi (50 nm)/LiF (1 nm)/Al (150 nm). After the UV−ozone treatment for 20 min, PEDOT:PSS spin-coated at 2000 rpm for 30 s on ITO and annealed at 150 °C for 20 min. Then transferred into an N2-filled glove box (<0.1 ppm O2 and H2O). 2PACz (0.5 mg mL−1 in EtOH) was deposited onto the PEDOT:PSS films with a spin-coating condition of 3000 rpm for 30 s, then annealed at 100 °C for 10 min. After the film cooled down to room temperature, mCP:target (6:4) (total concentration 10 mg mL−1 in CB and with 5% v/v anisole) was in the spin-coating condition of 1000 rpm, then annealed at 80 °C for 10 min. 50 nm TPBi, 1 nm LiF, and 150 nm Al electrode were evaporated sequentially under high vacuum (<1 × 10−6 Torr).

Characterization

The current density–voltage–radiance and Electroluminescence spectra characteristics were characterized by the LQ-50X system (Enlitech, Taiwan) includes a PTFE integrating sphere, a Multi-Channel Photon Detector (MCD) and two spectrometers to collect emission photons and subsequent spectral analyses. The MCD enhances sensitivity, facilitating effective detection in low-light conditions. The system is capable of measuring a broad wavelength range from 300 to 1700 nm using Si and InGaAs detectors, and it is calibrated against a NIST-traceable standard lamp. equipped with a source meter (Keithley 2400).

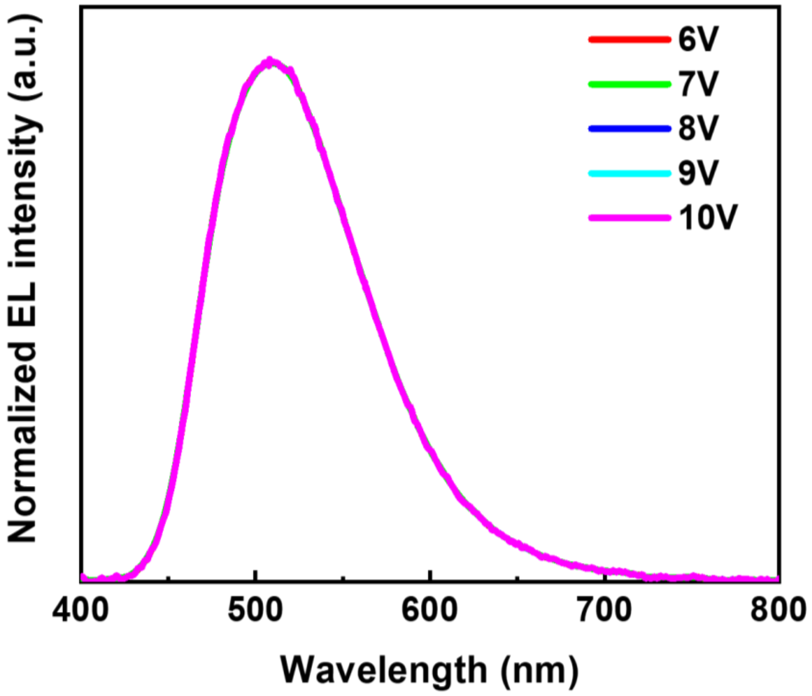

Figure S25. The normalized EL spectra of optimized devices (OLED of **CT-Rotaxane**) at different voltages.

The observed behavior can be attributed to partial leakage of compound **1** from the cage, which leads to an energy-level mismatch in the emitter layer. Therefore, a higher applied voltage is required to strengthen the internal electric field and facilitate electron–hole recombination. It is also noteworthy that the maximum luminance of **1@Trz-cage** reaches only ~30 cd m^{-2} (see Fig. S26), strongly indicating that exciton formation is predominantly driven by the intensified electric field rather than efficient radiative recombination under normal operating conditions.

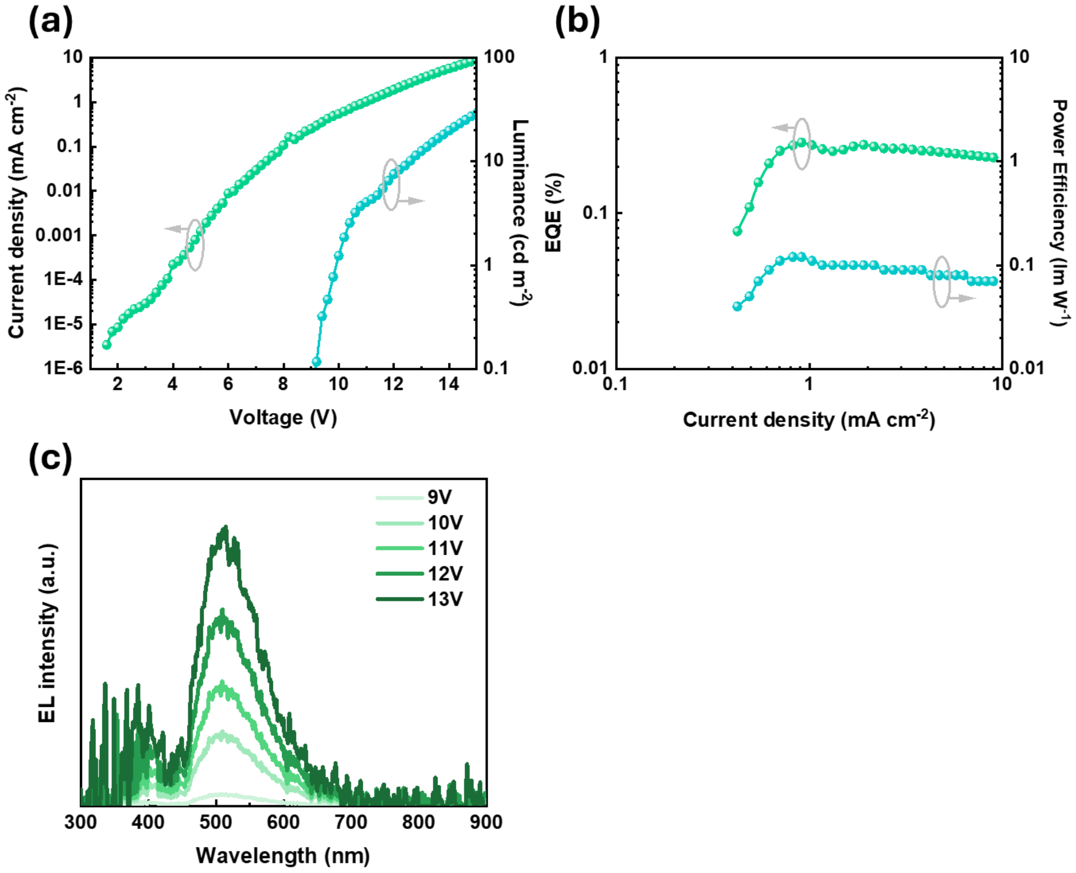

Figure S26. OLED device performances based on compound **1@Trz-cage** as an emitter. OLED performances based on **CT-Rotaxane** as an emitter. (a) Current (green line) and luminance (blue line) versus voltage curves. (b) EQE (green line) and power efficiency (blue line) versus current density curves. (c) EL spectra of optimized devices at different voltages.

7. NMR and MS Spectra

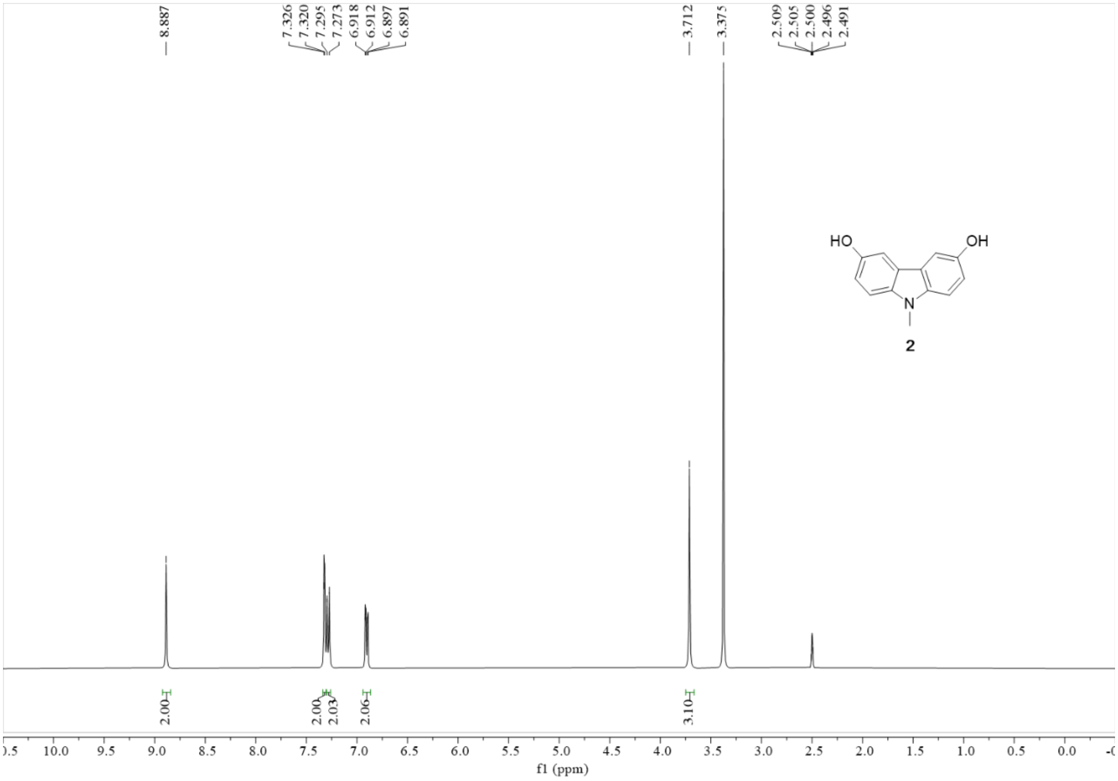

Figure S27. ^{1}H-NMR spectrum of **2**.

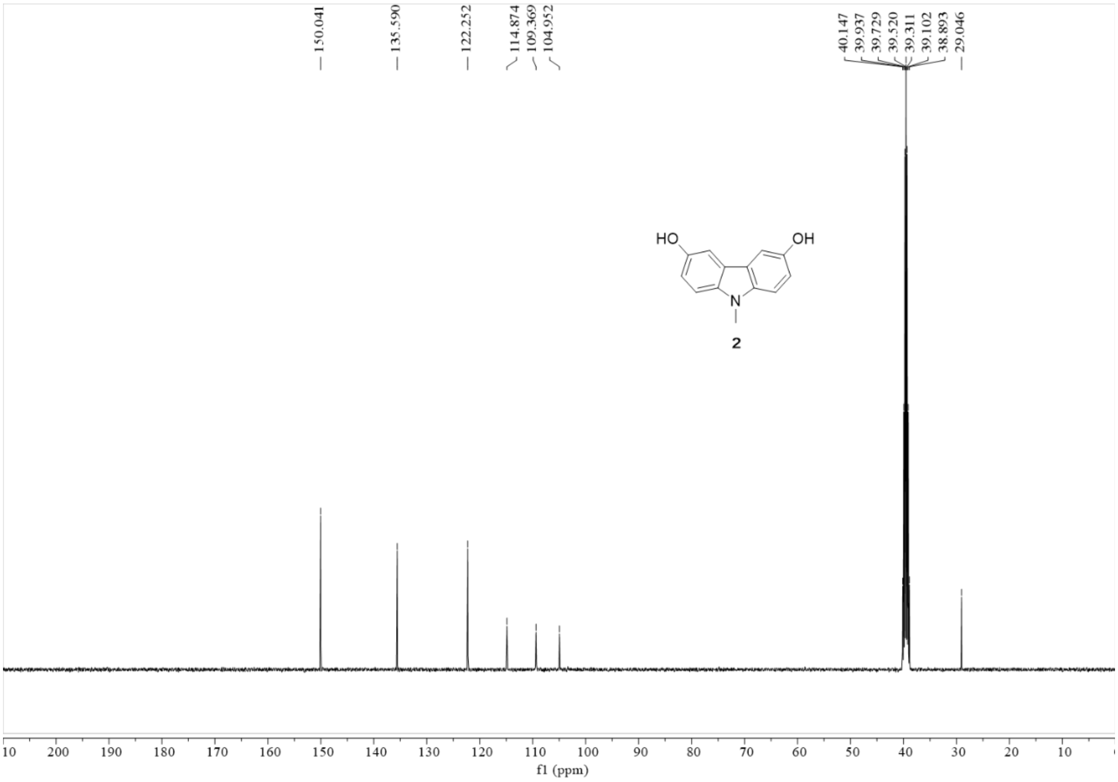

Figure S28. ^{13}C-NMR spectrum of **2**.

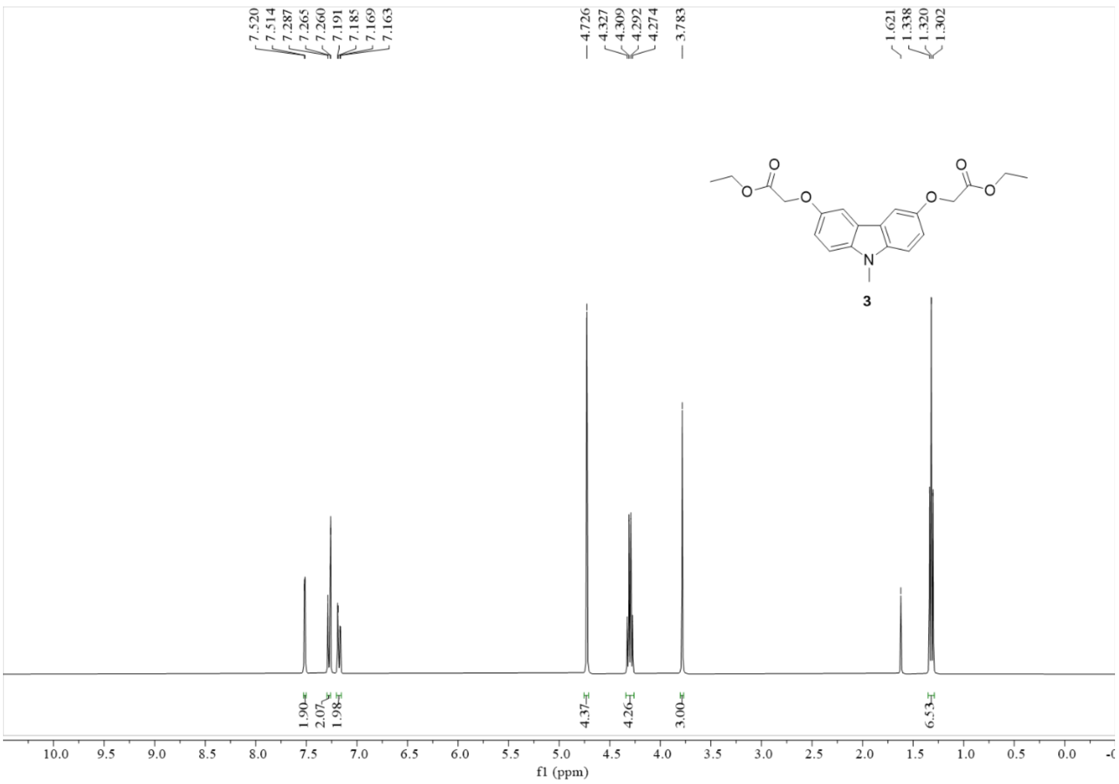

Figure S29. ^{1}H-NMR spectrum of **3**.

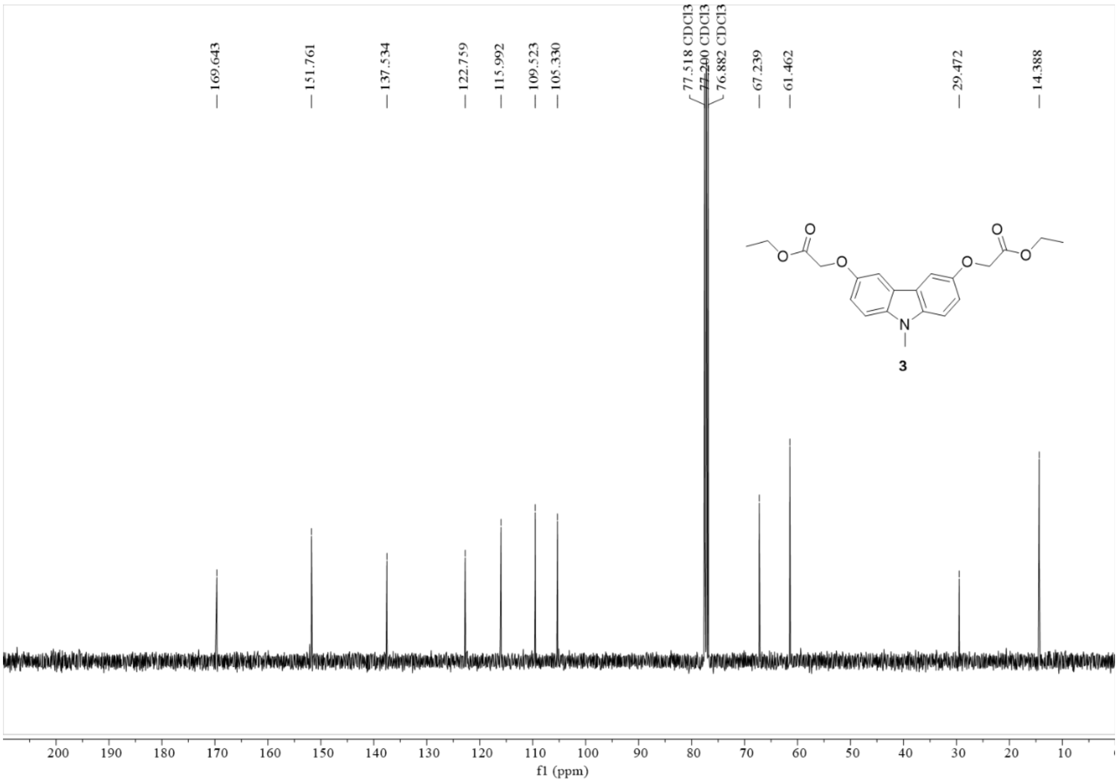

Figure S30. ^{13}C-NMR spectrum of **3**.

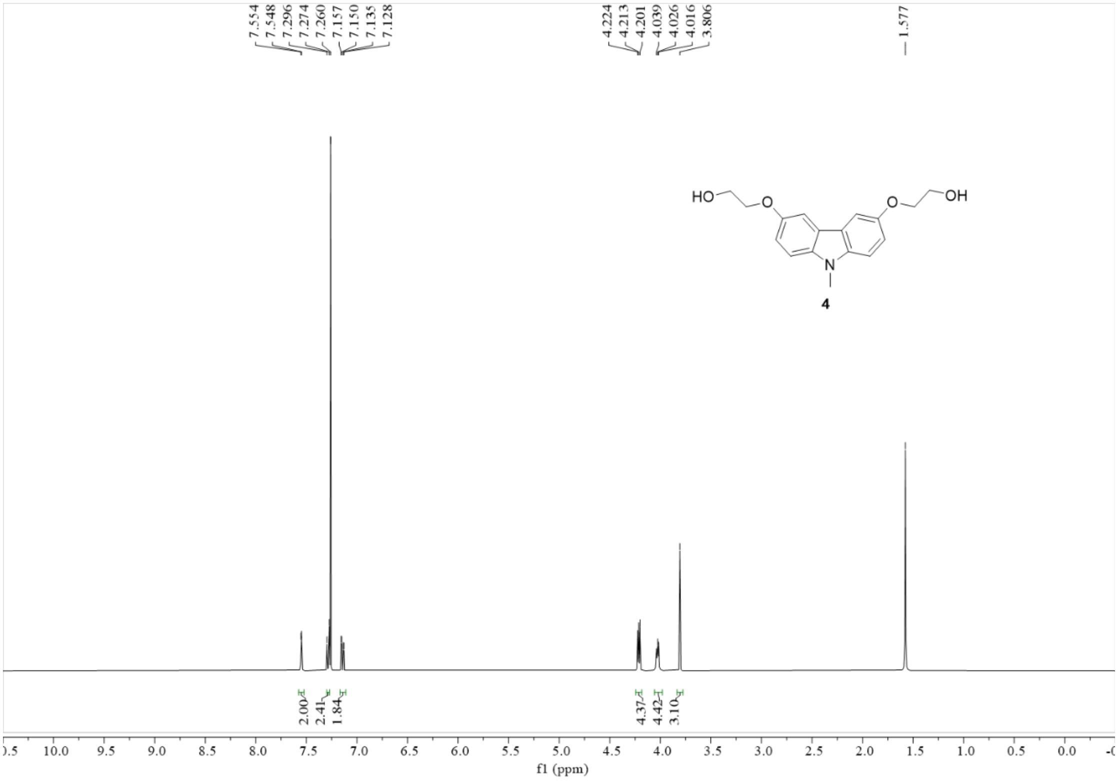

Figure S31. ^{1}H-NMR spectrum of **4**.

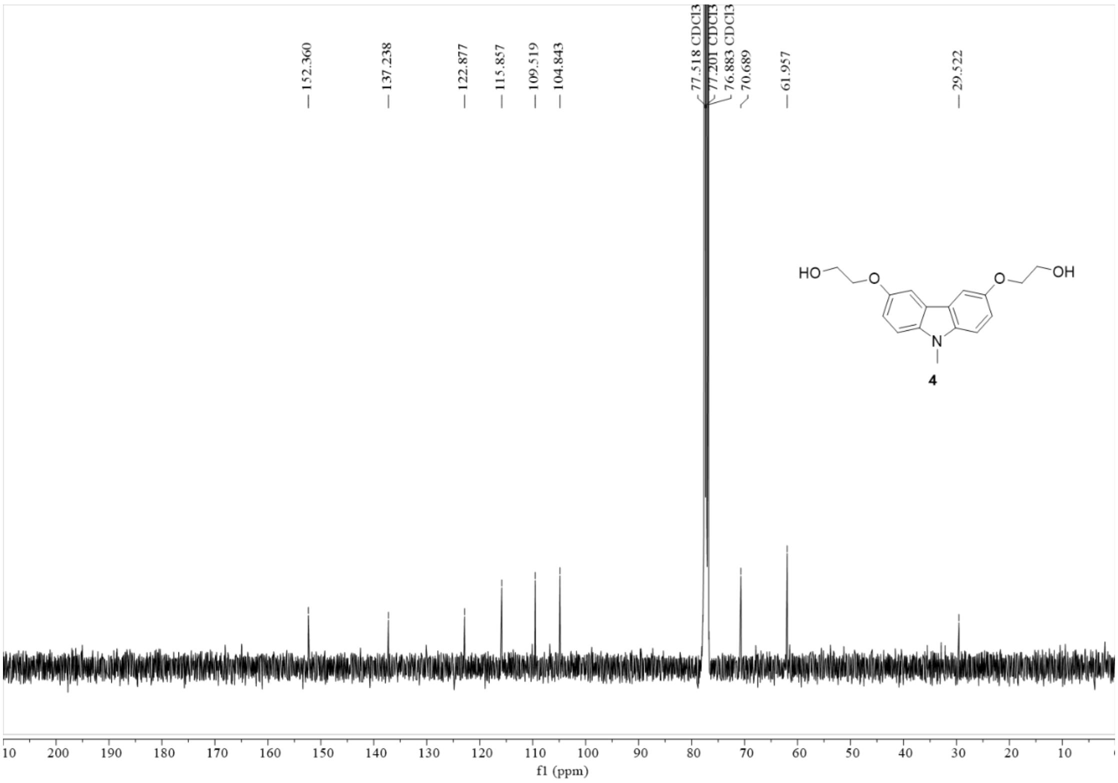

Figure S32. ^{13}C-NMR spectrum of **4**.

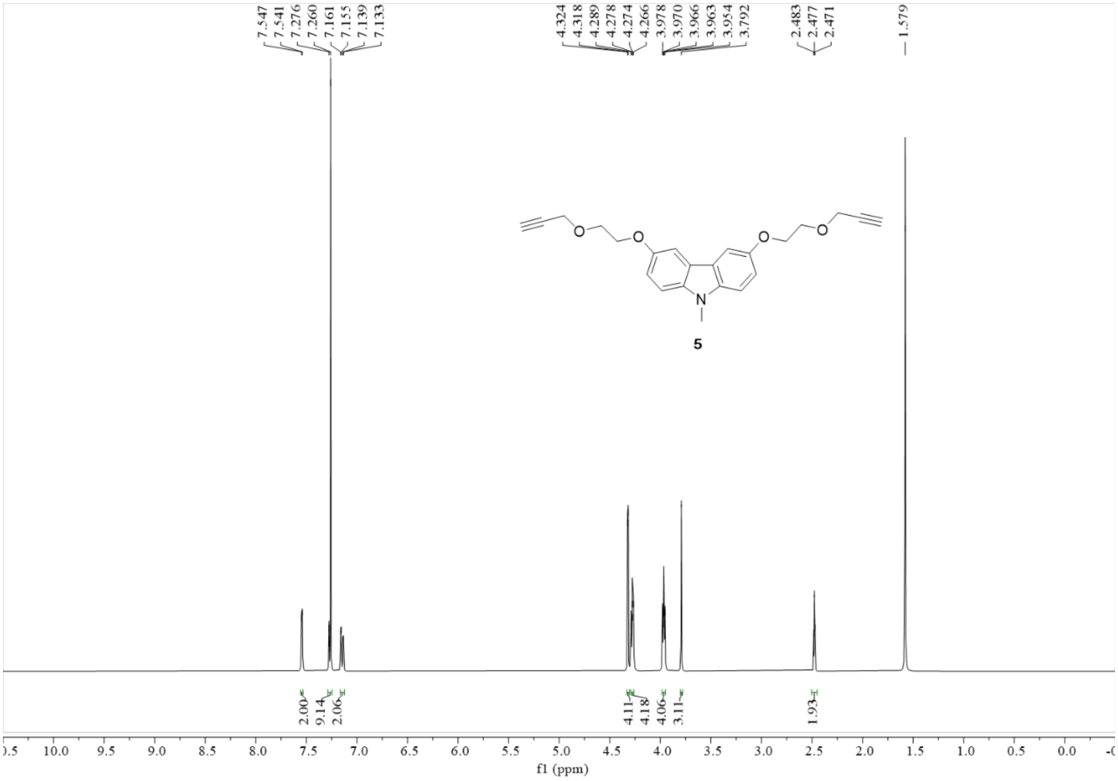

Figure S33. ^{1}H-NMR spectrum of **5**.

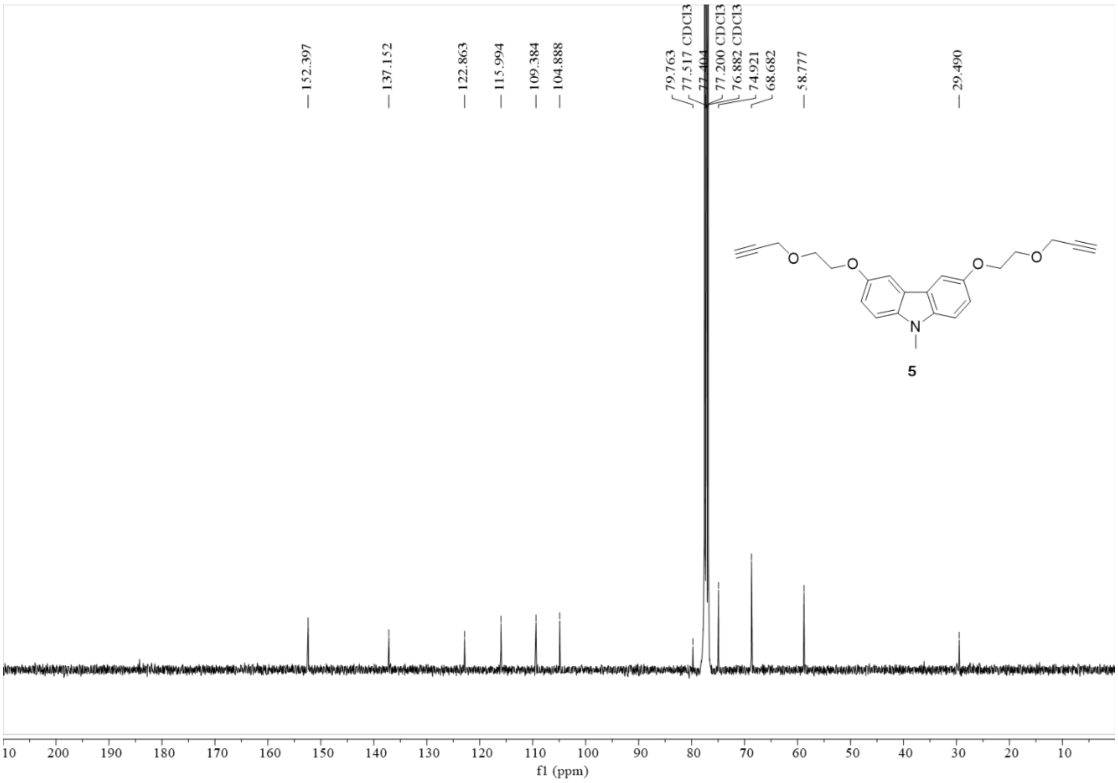

Figure S34. ^{13}C-NMR spectrum of **5**.

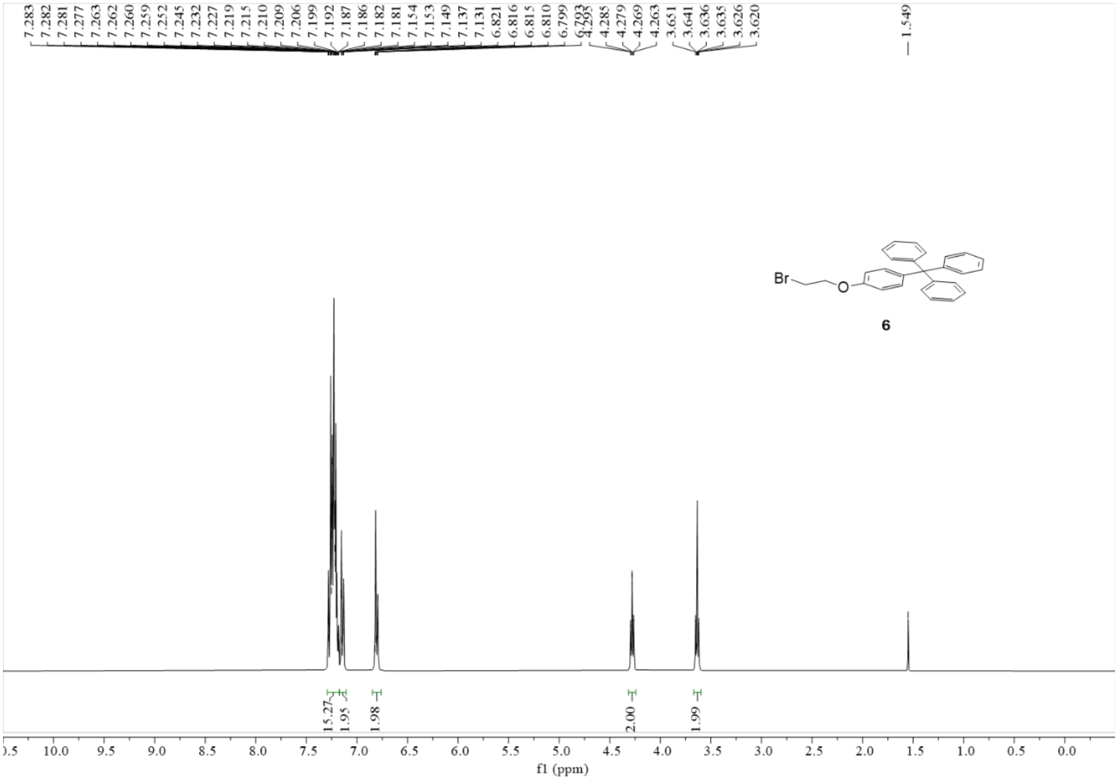

Figure S35. ^{1}H-NMR spectrum of **6**.

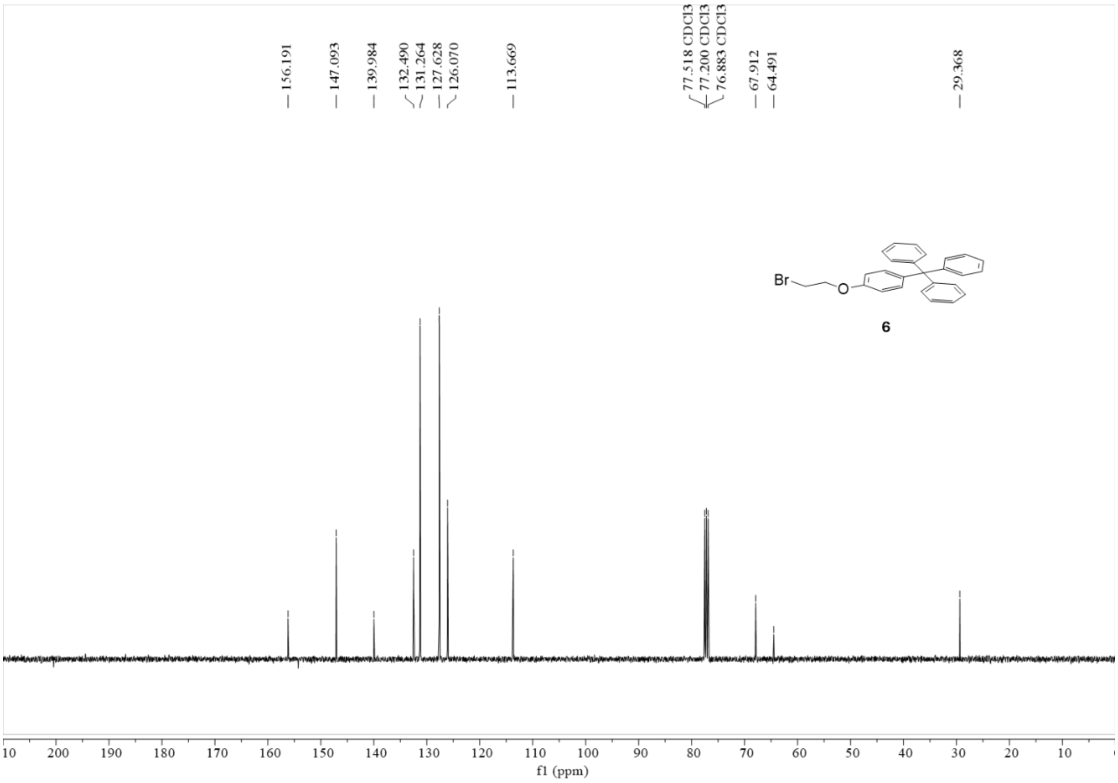

Figure S36. ^{13}C-NMR spectrum of **6**.

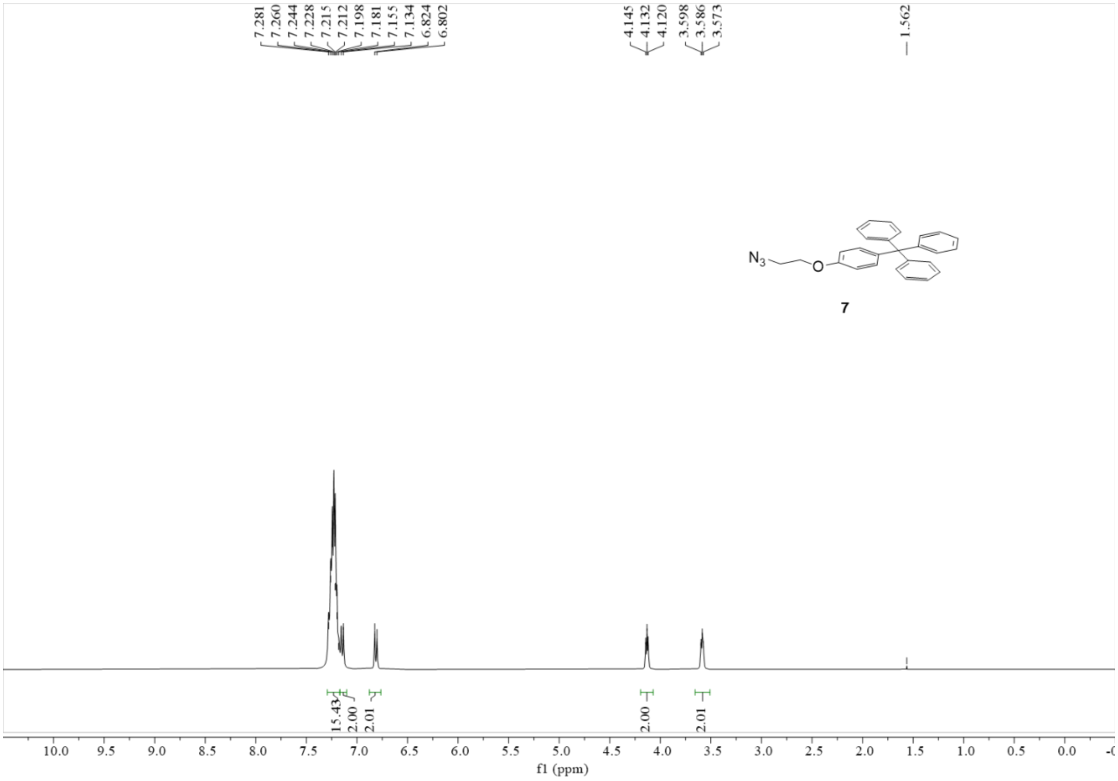

Figure S37. ^{1}H-NMR spectrum of **7**.

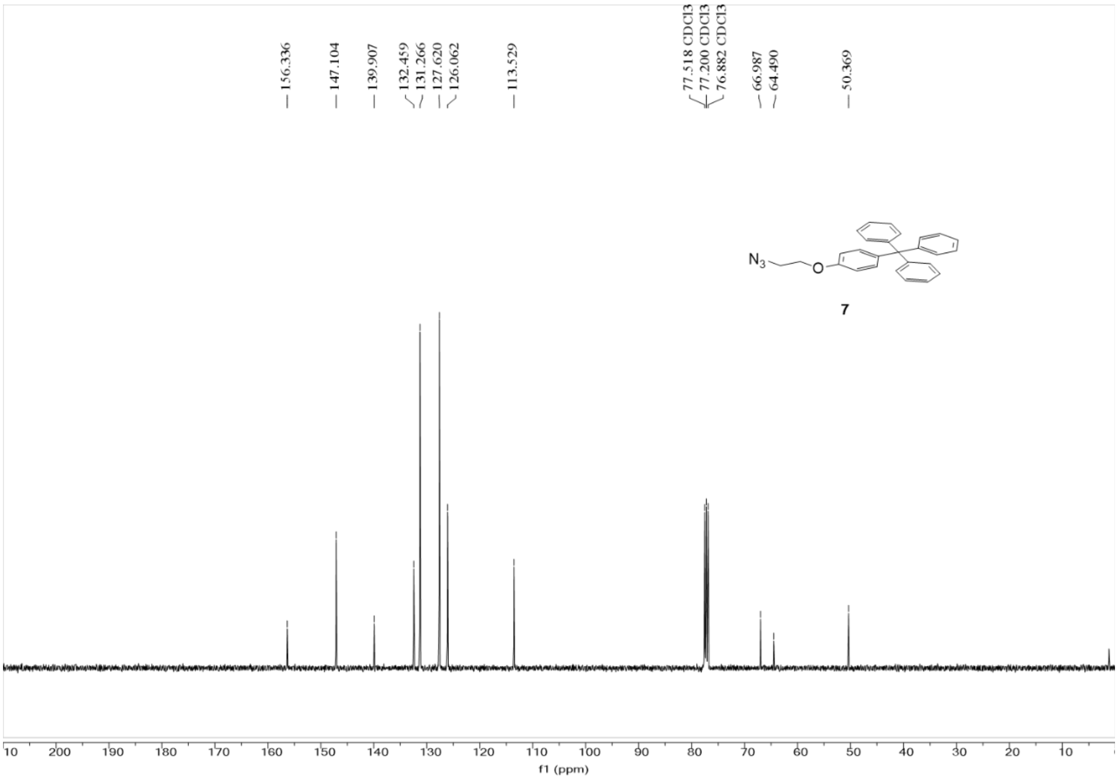

Figure S38. ^{13}C-NMR spectrum of **7**.

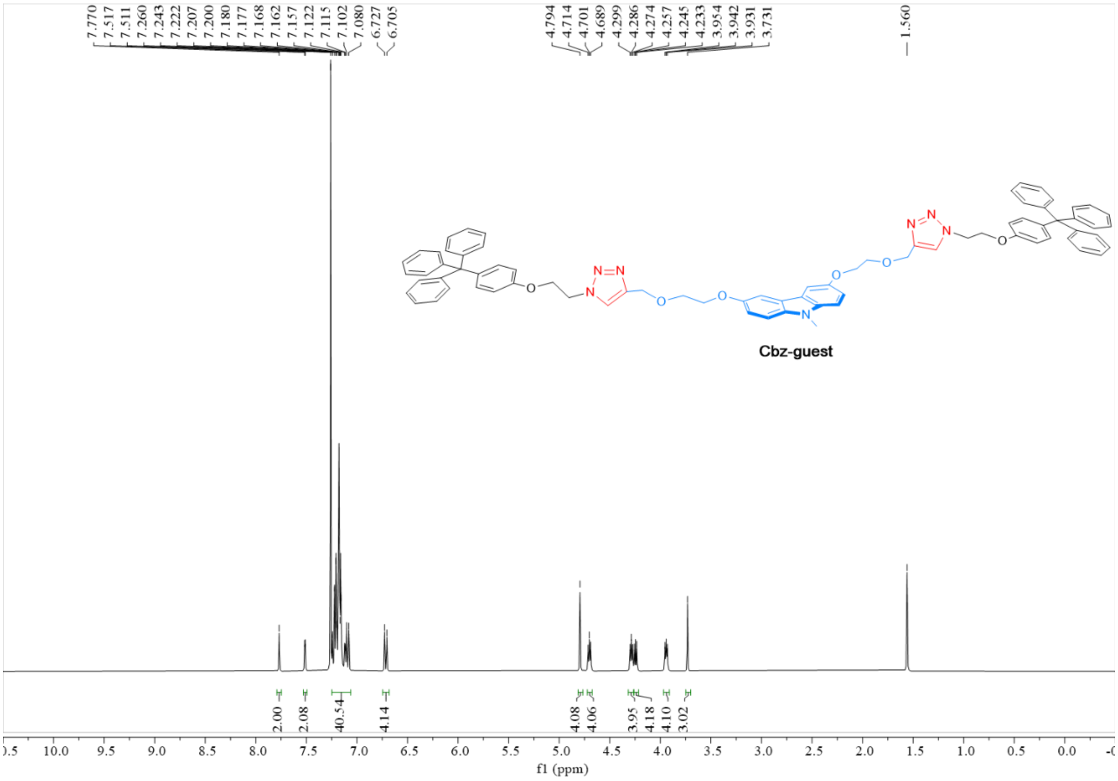

Figure S39. ^{1}H-NMR spectrum of **Cbz-guest**.

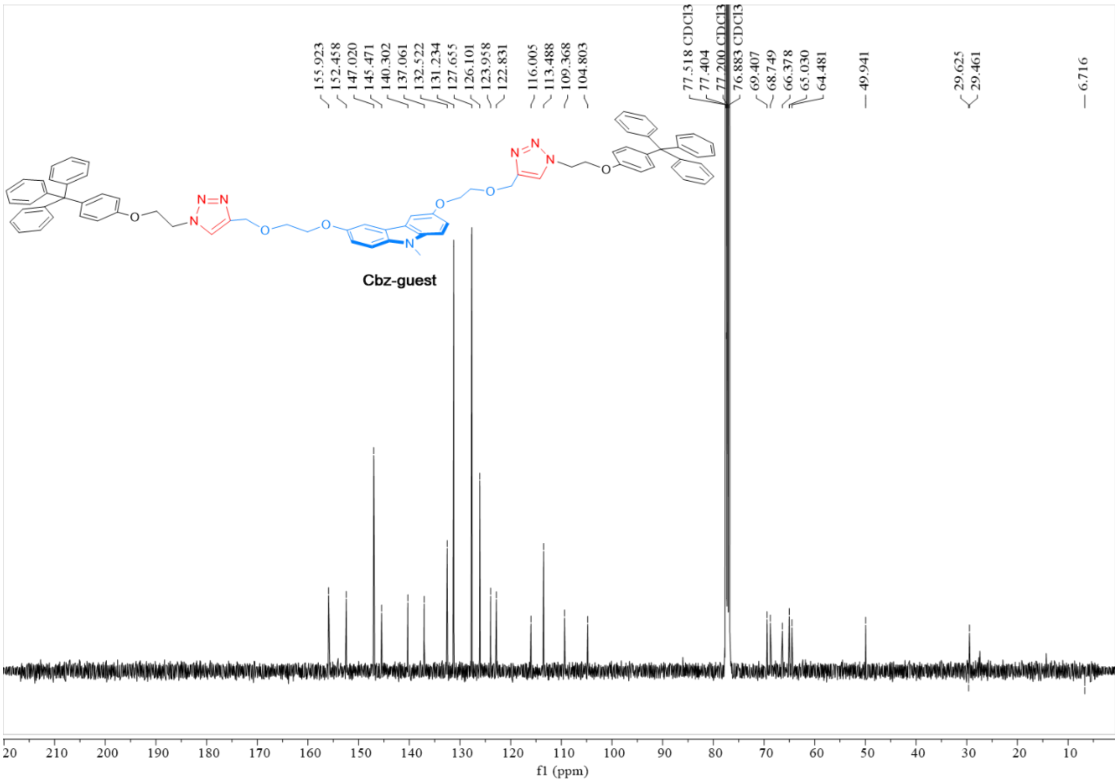

Figure S40. ^{13}C-NMR spectrum of **Cbz-guest**.

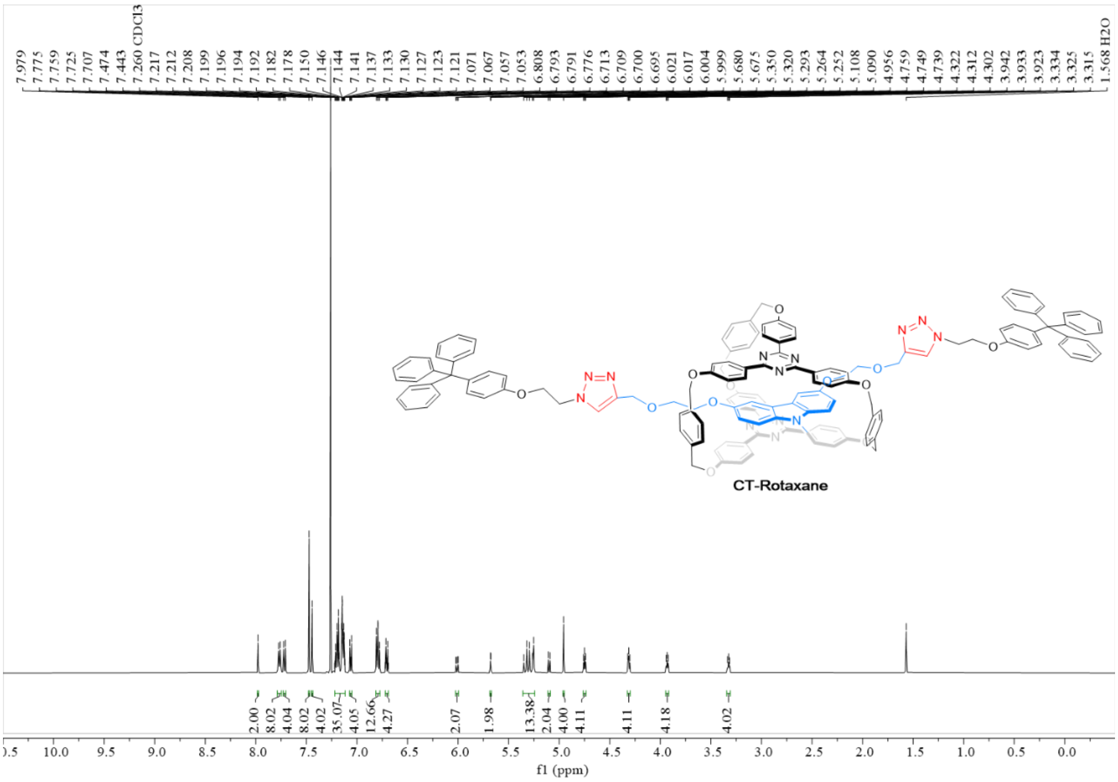

Figure S41. ^{1}H-NMR spectrum of **CT-Rotaxane**.

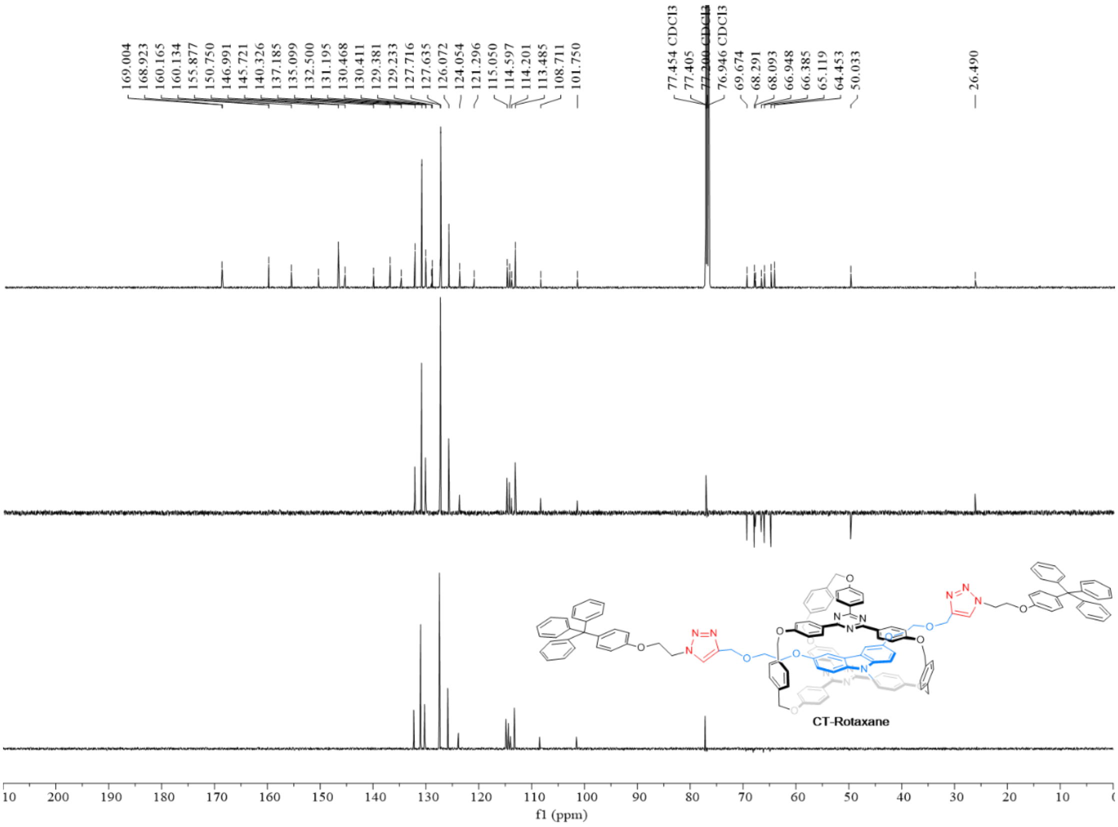

Figure S42. ^{13}C-NMR and DEPT spectrum of **CT-Rotaxane**.

8. References

[1]. Tsuchiya, Y. Diesing, S. Bencheikh, F. Wada, Y. dos Santos, P. L. Kaji, H. Zysman-Colman, E. Samuel, I. D. W. Adachi, C. Exact Solution of Kinetic Analysis for Thermally Activated Delayed Fluorescence Materials. *J. Phys. Chem. A* **2021**, *125*, 8074−8089.

[2]. Case, D. A.; Aktulga, H. M.; Belfon, K.; Ben-Shalom, I. Y.; Berryman, J. T.; Brozell, S. R.; Carvahol, F. S.; Cerutti, D. S.; Cheatham, I. T. E.; Cisneros, G. A.; Cruzeiro, V. W. D.; Darden, T. A.; Forouzesh, N.; Ghazimirsaeed, M.; Giambaşu, G.; Giese, T.; Gilson, M. K.; Gohlke, H.; Goetz, A. W.; Harris, J.; Huang, Z.; Izadi, S.; Izmailov, S. A.; Kasavajhala, K.; Kaymak, M. C.; Kolossváry, I.; Kovalenko, A.; Kurtzman, T.; Lee, T. S.; Li, P.; Li, Z.; Lin, C.; Liu, J.; Luchko, T.; Luo, R.; Machado, M.; Manathunga, M.; Merz, K. M.; Miao, Y.; Mikhailovskii, O.; Monard, G.; Nguyen, H.; O'Hearn, K. A.; Onufriev, A.; Pan, F.; Pantano, S.; Rahnamoun, A.; Roe, D. R.; Roitberg, A.; Sagui, C.; Schott-Verdugo, S.; Shajan, A.; Shen, J.; Simmerling, C. L.; Skrynnikov, N. R.; Smith, J.; Swails, J.; Walker, R. C.; Wang, J.; Wang, J.; Wu, X.; Wu, Y.; Xiong, Y.; Xue, Y.; York, D. M.; Zhao, C.; Zhu, Q.; Kollman, P. A., *Amber 2025*. University of California, San Francisco: 2025.

[3]. Martínez, L.; Andrade, R.; Birgin, E. G.; Martínez, J. M., PACKMOL: A package for building initial configurations for molecular dynamics simulations. *J. Comput.Chem.* **2009,** *30* (13), 2157-2164.

[4]. Wang, J.; Wolf, R. M.; Caldwell, J. W.; Kollman, P. A.; Case, D. A., Development and testing of a general amber force field. *J. Comput. Chem.* **2004,** *25* (9), 1157-1174.

[5]. Case, D. A.; Aktulga, H. M.; Belfon, K.; Cerutti, D. S.; Cisneros, G. A.; Cruzeiro, V. W. D.; Forouzesh, N.; Giese, T. J.; Götz, A. W.; Gohlke, H.; Izadi, S.; Kasavajhala, K.; Kaymak, M. C.; King, E.; Kurtzman, T.; Lee, T.-S.; Li, P.; Liu, J.; Luchko, T.; Luo, R.; Manathunga, M.; Machado, M. R.; Nguyen, H. M.; O'Hearn, K. A.; Onufriev, A. V.; Pan, F.; Pantano, S.; Qi, R.; Rahnamoun, A.; Risheh, A.; Schott-Verdugo, S.; Shajan, A.; Swails, J.; Wang, J.; Wei, H.; Wu, X.; Wu, Y.; Zhang, S.; Zhao, S.; Zhu, Q.; Cheatham, T. E., III; Roe, D. R.; Roitberg, A.; Simmerling, C.; York, D. M.; Nagan, M. C.; Merz, K. M., Jr., AmberTools. *Journal of Chemical Information and Modeling* **2023,** *63* (20), 6183-6191.

[6]. Frisch, M. J.; Trucks, G. W.; Schlegel, H. B.; Scuseria, G. E.; Robb, M. A.; Cheeseman, J. R.; Scalmani, G.; Barone, V.; Petersson, G. A.; Nakatsuji, H.; Li, X.; Caricato, M.; Marenich, A. V.; Bloino, J.; Janesko, B. G.; Gomperts, R.; Mennucci, B.; Hratchian, H. P.; Ortiz, J. V.; Izmaylov, A. F.; Sonnenberg, J. L.; Williams; Ding, F.; Lipparini, F.; Egidi, F.; Goings, J.; Peng, B.; Petrone, A.; Henderson, T.; Ranasinghe, D.; Zakrzewski, V. G.; Gao, J.; Rega, N.; Zheng, G.; Liang, W.; Hada, M.; Ehara, M.; Toyota, K.; Fukuda, R.; Hasegawa, J.; Ishida, M.; Nakajima, T.; Honda, Y.; Kitao, O.; Nakai, H.; Vreven, T.; Throssell, K.; Montgomery Jr., J. A.; Peralta, J. E.; Ogliaro, F.; Bearpark, M. J.; Heyd, J. J.; Brothers, E. N.; Kudin, K. N.; Staroverov, V. N.; Keith, T. A.; Kobayashi, R.; Normand, J.; Raghavachari, K.; Rendell, A. P.; Burant, J. C.; Iyengar, S. S.; Tomasi, J.; Cossi, M.; Millam, J. M.; Klene, M.; Adamo, C.; Cammi, R.; Ochterski, J. W.; Martin, R. L.; Morokuma, K.; Farkas, O.; Foresman, J. B.; Fox, D. J. *Gaussian 16 Rev. C.01*, Wallingford, CT, 2016.

[7]. Lu, T. A comprehensive electron wavefunction analysis toolbox for chemists, Multiwfn. *J. Chem. Phys.* **2024** *161*, 082503.

[8]. Lu, T.; Chen, F. Multiwfn: A multifunctional wavefunction analyzer *J. Comput. Chem.* **2012**, *33* (5), 580–592.
